# APE1/Ref-1 Role in Inflammation and Immune Response

**DOI:** 10.3389/fimmu.2022.793096

**Published:** 2022-02-28

**Authors:** Thais Teixeira Oliveira, Leonam Gomes Coutinho, Laysa Ohana Alves de Oliveira, Ana Rafaela de Souza Timoteo, Guilherme Cavalcanti Farias, Lucymara Fassarella Agnez-Lima

**Affiliations:** ^1^ Departamento de Biologia Celular e Genética, Universidade Federal do Rio Grande do Norte (UFRN), Natal, Brazil; ^2^ Instituto Federal de Educação, Ciência e Tecnologia do Rio Grande do Norte (IFRN), São Paulo do Potengi, Brazil

**Keywords:** cytokines, NF-κB, biomarker, innate immunity, DNA repair, inflammation, reactive oxygen species, oxidized DNA damage

## Abstract

Apurinic/apyrimidinic endonuclease 1/redox effector factor 1 (APE1/Ref-1) is a multifunctional enzyme that is essential for maintaining cellular homeostasis. APE1 is the major apurinic/apyrimidinic endonuclease in the base excision repair pathway and acts as a redox-dependent regulator of several transcription factors, including NF-κB, AP-1, HIF-1α, and STAT3. These functions render APE1 vital to regulating cell signaling, senescence, and inflammatory pathways. In addition to regulating cytokine and chemokine expression through activation of redox sensitive transcription factors, APE1 participates in other critical processes in the immune response, including production of reactive oxygen species and class switch recombination. Furthermore, through participation in active chromatin demethylation, the repair function of APE1 also regulates transcription of some genes, including cytokines such as TNFα. The multiple functions of APE1 make it an essential regulator of the pathogenesis of several diseases, including cancer and neurological disorders. Therefore, APE1 inhibitors have therapeutic potential. APE1 is highly expressed in the central nervous system (CNS) and participates in tissue homeostasis, and its roles in neurodegenerative and neuroinflammatory diseases have been elucidated. This review discusses known roles of APE1 in innate and adaptive immunity, especially in the CNS, recent evidence of a role in the extracellular environment, and the therapeutic potential of APE1 inhibitors in infectious/immune diseases.

## Introduction

### APE1 - From Structure to Function

Apurinic/apyrimidinic endonuclease 1/Redox Factor-1 (APE1/Ref-1) is a multifunctional 35.6 kDa protein that responds to DNA damage (primarily DNA damage caused by oxidative stress) (1, 2). The C-terminal domain of APE1 processes apurinic/apyrimidinic (AP) sites generated by DNA glycosylase in the base excision repair (BER) pathway. The AP endonuclease activity of APE1 hydrolyzes the phosphodiester bond at these sites, generating a 3′-hydroxyl end (3′-OH) and a 5′-deoxyribose phosphate (5′-dRP) terminus. DNA polymerase β (Polβ) then removes the 5′-dRP and inserts the correct nucleotide. DNA ligase IIIα in complex with XRCC1 seals the phosphodiester bond, terminating the BER pathway. Occasionally, several nucleotides are removed by other enzymes through a sub-pathway known as long patch repair (1, 3, 4). Although other endonucleases act in the BER pathway, APE1 is the major AP endonuclease that repairs damage caused by oxidative stress, maintaining genome integrity in mammals (2, 5).

**Figure 1:**
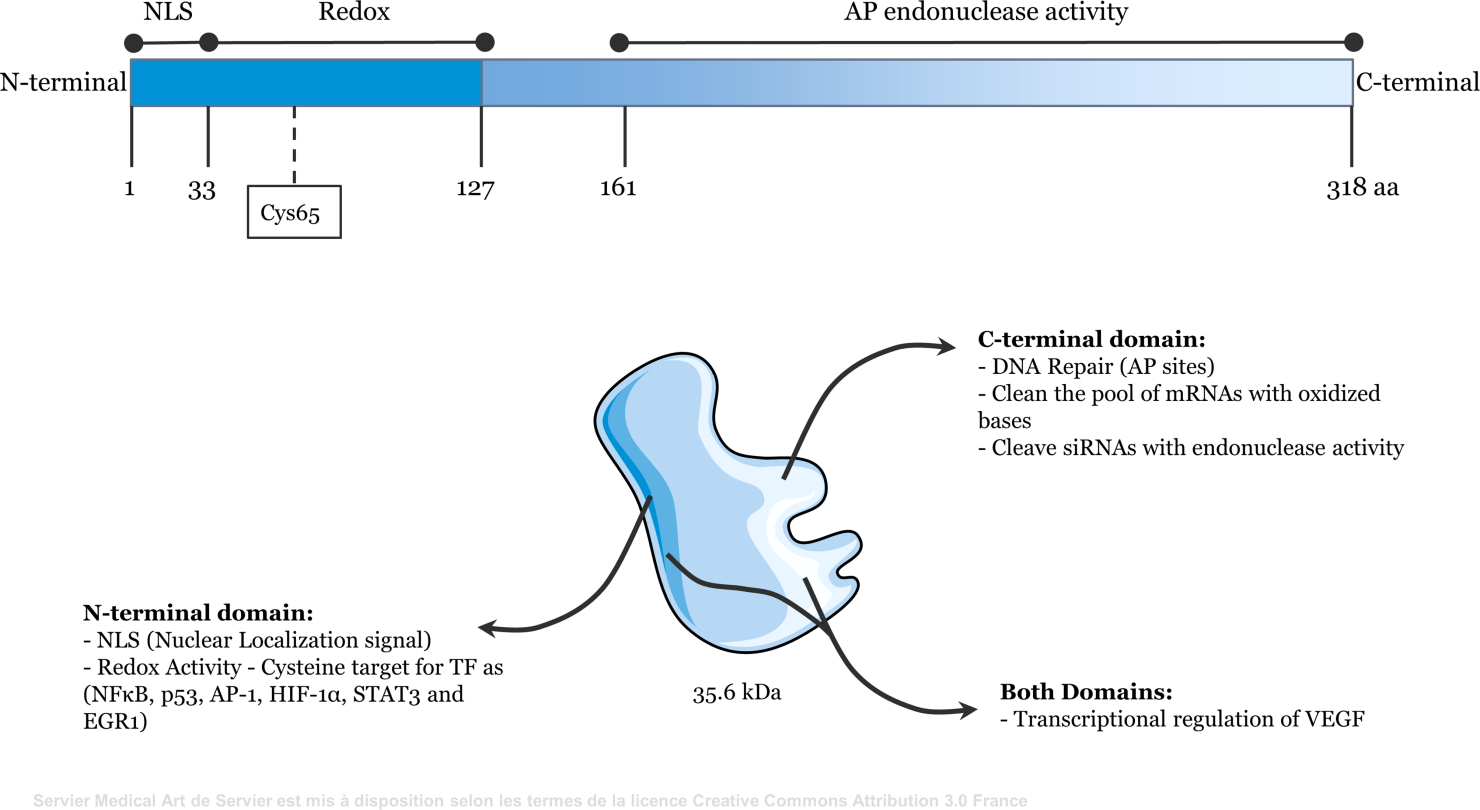
Representative scheme of the functional domains of Apurinic/apyrimidinic endonuclease 1/redox effector factor 1 (APE1/Ref-1). The N-terminal domain (amino acids 1 to 127) contains redox activity and the nuclear localization signal. The C-terminal domain (amino acids 161 to 318) contains apurinic/apyrimidinic endonuclease activity. Both domains may be involved in the transcriptional regulation of some genes, such as VEGF.

The N-terminal domain of APE1 has redox activity and contains a nuclear localization signal in its first 33 amino acids. APE1 reduces the cysteine ​​residues of target transcription factors (TFs) through exchange of protons with cysteine ​​residues present in its N-terminal region (6). The functional domains of APE1 are shown in **Figure 1**. The redox function of APE1 activates TFs, such as NF-κB, p53, activator protein 1 (AP-1), hypoxia-inducible factor-1α (HIF-1α), signal transducer and activator of transcription 3 (STAT3), and early growth response 1 (EGR1) (7–12). Therefore, APE1 regulates the expression of genes that directly affect several cellular processes, including inflammatory responses (13, 14). For example, APE1 reduces HIF-1α, increasing its DNA-binding activity. This induces expression of vascular endothelial growth factor (VEGF), which promotes angiogenesis (15–17). Additionally, because APE1 regulates STAT3, NF-κB, EGR1, and AP-1, it directly influences the immune system by regulating the expression of cytokines and chemokines, including tumor necrosis factor alpha (TNFα), interleukin (IL)-6, and IL-8 (18–21). APE1 also interacts with ERK2 rescuing ERK2 from oxidative inactivation through its redox activity (22). The MEK-ERK1/2 pathway is a critical regulator of lipopolysaccharide (LPS)-induced responses (23).

The DNA repair activity of APE1 has recently been observed to play a role in transcriptional regulation. 8-oxoguanine (8-oxoG) is the most frequent DNA lesion caused by oxidative stress (24). This lesion is removed by 8-oxoguanine glycosylase (OGG1) and APE1 in the BER pathway. The presence of 8-oxoG can delay RNA polymerase progression, inducing transcriptional arrest and initiating DNA repair. Thus, 8-oxoG functions as a repressor in transcriptional regulation of genes (25). Some studies suggest that 8-oxoG can function as an epigenetic signal that favors the expression of several genes (26–29). Pan et al. observed that TNFα treatment induces an increase in 8-oxoG and OGG1 binding in promoters of proinflammatory genes, stimulating NF-κB binding to these sites, leading to gene activation and cytokine expression (27). Corroborating these data, 8-oxoG generation in G-quadruplex promoter sites favors OGG1 recruitment, generating AP sites that are substrates of APE1. The presence of APE1 in these promoters leads to TF recruitment and gene activation (30). Similarly, demethylation of histone H3, mediated by lysine-specific histone demethylase 1A, produces H_2_O_2_, leading to formation of local 8-oxoG lesions. Occurrence of 8-oxoG, and recruitment of OGG1 and APE1 have been observed to enhance the DNA-binding activity of MYC to its target gene promoters, thereby increasing gene expression (31).

Enzymes of the BER pathway, including APE1, are associated with active chromatin demethylation. This process is initiated by oxidation of 5-methylcytosines by ten-eleven translocation (TET). This oxidized base is removed by glycosylases such as thymine-DNA glycosylase (TDG), which generate AP sites. Thus, the endonuclease activity of APE1 is also involved in regulating chromatin and gene expression (24–27) [reviewed in (32)]. Thus, some genes, including VEGF, and cytokines, are regulated by the redox and repair functions of APE1 (33, 34).

In addition to regulating TF activity and maintaining genomic stability through DNA damage repair, APE1 plays an essential role in cell senescence by maintaining telomere stability and size through interaction with the telomere-protective proteins TRF1 and TRF2, and with POT1 (35, 36). APE1 also processes mRNAs that contain oxidized bases, thus preventing abnormal protein synthesis (37, 38). In addition to regulating mRNAs, APE1 can cleave siRNAs *in vitro* (38). APE1 has also been linked to numerous pathological processes, owing to its multiple functions in cellular homeostasis. APE1 is frequently overexpressed in cancer cells and is associated with increased resistance to chemotherapy (39). APE1 participates in signaling pathways involved in immune and inflammatory responses, which regulate gene expression of several innate and adaptive immune system mediators and is also involved in antibody production. In the following sections, the roles of APE1 and its functions in the immune system are described.

## APE1 in Innate Immunity

The mammalian immune system is divided into innate and adaptive systems. The innate immune system recognizes pathogen-associated molecular patterns (PAMPs) and damage-associated molecular patterns (DAMPs) through germline-encoded receptors, such as pattern recognition receptors (PRRs). In the presence of PAMPs and DAMPs, cells of the innate immune system initiate an acute inflammatory response by secreting cytokines, chemokines, reactive oxidative species (ROS), and other inflammatory mediators to attract immune cells to the site of damage (40–42). ROS production plays a central role in inflammatory signaling by eliminating pathogens in phagocytic cells or acting as signaling molecules. ROS are endogenously produced in the mitochondria, peroxisomes, and endoplasmic reticulum, and by NADPH oxidases (NOX) in phagocytes and endothelial cells (42, 43). Chronic ROS exposure or imbalance between ROS and antioxidants plays a critical role in the progression of inflammatory diseases, including inflammatory bowel disease (44, 45), hepatitis (46), atherosclerosis (47), and multiple sclerosis (48).

At least two highly interconnected ROS-related processes occur in the innate immune system. First, ROS can induce an inflammatory response leading to APE1 expression. Several studies have shown that ROS induces APE1 expression and activity in different cell types, including macrophages and human gastric epithelial cell lines infected with *Helicobacter pylori* (49–51). In these cells, *H. pylori* infection and TNFα treatment induced activation of NF-κB, and AP-1 and IL-8 expression were inhibited by APE1 silencing (52, 53). APE1 inhibition also prevented H_2_O_2_-induced increase in IL-6 and IL-4 expression in mast cells (54). Antioxidant enzymes, such as Peroxiredoxin 1, also appear to regulate cytokine and chemokine expression in an APE1-dependent manner. Nassour et al. reported that APE1 interacts with Peroxiredoxin 1 in HeLa cells under physiological conditions or with H_2_O_2_ treatment. This interaction may prevent APE1 from reducing TFs, including NF-κB, and decrease IL-8 expression, attributing an APE1-dependent anti-inflammatory role to Peroxiredoxin 1 (21).

Second, the inflammatory response can induce ROS production. For example, APE1-deficient human cells infected with *H. pylori* show high Rac1 activation and NOX1 expression. Consistent with these findings, APE1 overexpression decreased ROS levels, Rac1 activation, and NOX1 expression in *H. pylori-*infected cells. APE1, through its N-terminal lysine residues, interacts with Rac1, decreasing NOX1 expression and ROS generation (55). Therefore, APE1 appears to have an inhibitory effect on ROS production. Granzyme K is a tryptase that is highly expressed in natural killer (NK) cells and is necessary for NK cell-mediated cytolysis. Granzyme K -mediated apoptosis is initiated by ROS accumulation and cytochrome C release (56). Granzyme K cleaves APE1, abrogating its antioxidant activity (57). The resultant decrease in APE1 levels correlates with NK cell-mediated apoptosis of tumor cells or virus-infected cells, indicating that APE1 is essential for maintaining cell viability.

Cytokine- and chemokine-mediated signaling is involved in both innate and adaptive immunity. These signaling molecules are often transcriptionally regulated by TFs, including NF-κB, AP-1, EGR1, and STAT3, which are activated by the redox function of APE1, which in turn increases their DNA-binding capacity (11, 12, 58). APE1 exerts a proinflammatory role in stimulating cytokine and chemokine expression. APE1 knockdown in keratinocytes treated with synthetic lipopeptide or zymosan resulted in decreased NF-κB activation and TNFα and IL-8 expression (18). Treatment of macrophages with APE1 redox inhibitor E3330 decreases NF-κB and AP-1 activation, and consequently, TNFα, IL-6, IL-12, PGE2, and COX-2 expression (19). E3330 also inhibits IL-8 expression in TNFα-induced JHH6 cells (59). E3330 (also called APX3330) is a quinone derivative and a specific inhibitor of APE1 redox functions (60–62). E3330 acts by binding to APE1 and increasing the formation of disulfide bonds between cysteine residues (Cys65 or Cys93) which are critical for redox function (61, 63), without affecting AP endonuclease activity (60–62, 64). In addition, in a gastric inflammation model of *H. pylori*, APE1 redox inhibition reduced cytokine expression, decreased immune cell infiltration, and exerted neuroprotective effects on the enteric nervous system (65). These studies demonstrate the role of APE1 redox activity as a positive regulator of cytokine and chemokine expression in innate immune system cells.

Many studies have demonstrated dual roles of APE1 in inflammation. Ectopic APE1 overexpression appears to play an anti-inflammatory role in some cells. In the macrophage-like THP-1 cell line, APE1 transfection decreased the expression of IL-6, TNFα, and IL-1 induced by oxidized LDL (66) and TNFα and COX-2 expression induced by HMGB1 (67). In addition, using the *APE1* gene cloned in an expression vector and administered *via* retrograde renal vein injection, Maruyama et al. demonstrated that APE1 expression inhibits the development of tubulointerstitial fibrosis and modulates the immune system through different pathways, including IL-6, TNFα, and IL-1β (68). The dual role of APE1 in cytokine and chemokine expression can be attributed to specific functions of APE1 in different cell types. Yuk et al. observed a contradictory effect in the same cell line (THP-1) by analyzing the effects of APE1 overexpression and siRNA knockdown on HMGB1-induced inflammatory responses. The authors observed that siRNA-mediated inhibition decreased APE1 nuclear and cytoplasmic expression and impaired HMGB1-mediated cytokine expression and MAPK pathway activation. Furthermore, APE1 overexpression by adenoviral vectors has been reported to increase cytoplasmic APE1 expression, leading to a decrease in ROS levels, cytokine secretion, and cyclooxygenase-2 expression. A reduction in p38 and c-Jun N-terminal kinase activation and extracellular release of HMGB1 has also been observed (67). The authors suggested that APE1 compartmentalization may explain the contrasting functions described above (67). The role of cytoplasmic APE1 in the inflammatory process remains to be clarified.

APE1 deficiency in mice is associated with increased expression of inflammatory mediators in senescent cells. APE1 deficiency is also associated with decreases in the size of several organs including the brain (14). These events may be associated with senescence-associated secretory phenotype stimulation, which includes changes in the cell protein secretion profile, such as proinflammatory cytokines (IL-1α and IL-6), chemokines (IL-8), and growth factors (VEGF), many of which are regulated by the redox function of APE1 (69).

NF-κB and AP-1 are the main TFs observed in studies of APE1 expression or inhibition in inflammatory models. However, several TFs interact with the APE1 redox region. Occasionally, these factors also play a role in the inflammatory response or immunity. For example, HIF-1α, a classical target of APE1 redox activity (15, 16),is essential in glycolysis and angiogenesis. HIF-1α also participates in the immune response, and its inactivation decreases macrophage invasion, aggregation, and motility (70). APE1 redox function also regulates STAT3 transcriptional activity (7), affecting dendritic cell maturation and anti-inflammatory signaling in phagocytes and inflammatory responses related to cancer (71, 72). **Table 1** lists the principal TFs regulated by APE1, their functions, and studies reporting their involvement in the immune response.

**Table 1:** Transcription factors regulated by apurinic/apyrimidinic endonuclease 1/redox effector factor 1 (APE1/Ref-1) and their functions in the immune response.

TFs	Immune system function	Function inhibited	Refs
NF-κB	Inflammation, immunity, differentiation, cell growth, tumorigenesis, and apoptosis	Redox and repair	(10, 27)
AP-1	Proliferation, differentiation, and apoptosis	Redox	(11)
STAT3	Dendritic cells maturation, activation, and anti-inflammatory signalization in phagocytes	Redox	(7, 72)
HIF-1	Invasion, aggregation and motility of macrophages, and energy homeostasis	Redox and repair	(16, 33, 73)
EGR1	Differentiation of myeloid cells	Redox	(74, 75)
P53	Apoptosis, antiviral defense, induction of type I IFN, enhanced pathogen recognition, and immune checkpoint regulation	Redox and redox-independent functions	(76–78)
PAX5	B lymphopoiesis	Redox	(79, 80)
PTEN	DC maturation and T cell polarization	–	(76, 81)

In general, the studies cited above describe the associations of APE1 redox function or reduced APE1 expression with inflammatory regulation. However, our group recently showed that inhibition of AP site repair by methoxyamine inhibits the expression of IL-8, IL-6, TNFα, IL-10, and MCP1 in LPS-induced U937 cells (34). This treatment also decreased expression of genes involved in prostaglandin biosynthesis and MyD88-independent toll-like receptor signaling pathway genes. Reduced ELK1 expression after chemical inhibition of APE1 by E3330 or methoxyamine was also observed. ELK1 expression is regulated by ERK pathway, EGR1, and TET enzymes (82–84). In this context, our findings suggest that both redox and DNA repair activities of APE1 regulate ELK1 expression through independent but overlapping mechanisms (34). Together, these data suggest a role of DNA repair in regulating gene expression, influencing the expression of inflammatory mediators.

**Figure 2:**
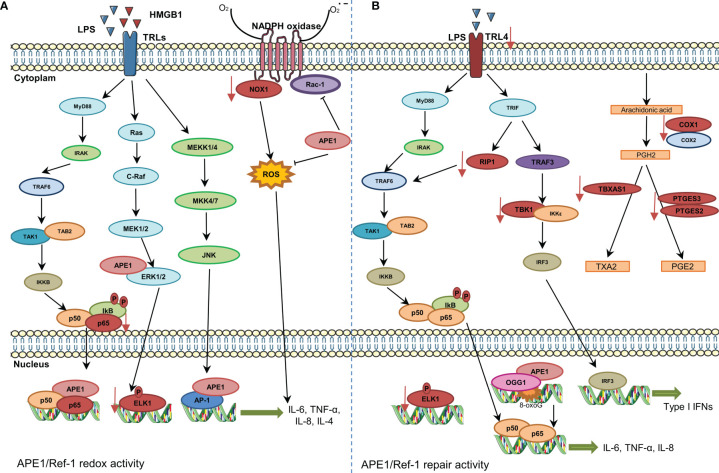
Involvement of APE1 in expression of cytokines and chemokines and reactive oxygen species (ROS) regulation. **(A)** The stimulation of Toll-like receptors promotes NF-κB activation and its translocation to the nucleus. APE1 redox function reduces transcription factors, such as NF-κB and AP-1, and increases expression of cytokines. APE1 also inhibits Rac1 and ROS production by NADPH oxidase. Inhibition of APE1 redox function decreases the expression of NOX1, P65, and ELK1 (represented in red color). **(B)** The DNA repair activity of APE1 also regulates expression of cytokines. 8-Oxoguanine DNA glycosylase and APE1 recruitment to damaged sites is essential for downstream recruitment of transcription factors. Additionally, inhibition of APE1 DNA repair activity decreases the expression of various genes/proteins (represented in red color).

**Figure 2** summarizes the main roles of APE1 in the inflammatory response. The potential of this response to be cell type-specific must be considered. Therefore, more studies are required to better understand the role of APE1 repair activity in the transcriptional regulation of proinflammatory genes.

## APE1 in Neuroinflammation

Increased APE1 expression in the nervous system is well-documented (85–87). APE1 expression varies in different tissues under normal physiological conditions. High APE1 levels are observed in the dentate gyrus granule cells, cerebellar Purkinje cells, and piriform cortex neurons (85). However, APE1 expression is significantly increased in the brain and spinal cord of individuals affected by diseases including amyotrophic lateral sclerosis (ALS), compared to healthy controls (88). Excessive ROS production in neurons in response to certain stimuli is associated with APE1 expression (89). ROS originate from many sources but have mainly been attributed to high mitochondrial respiration activity or malfunctioning organelles. Thus, many studies have focused on neuronal mitochondrial dysfunction during ischemia to assess the role of APE1 (90, 91).

APE1 upregulation generally protects neuronal structure and function during transient global cerebral ischemia (90, 91). This protection has been mainly attributed to its role in BER, which corrects damage induced by ROS. Although BER is the predominant mechanism for repairing oxidized DNA damage in neurons, APE1 also participates in non-homologous end-joining repair mechanisms in cortical neurons (92). In addition, APE1 helps regulate the nucleotide excision repair pathway to repair DNA adducts induced by cisplatin in sensory neurons (93).

Oxidative stress in neurons plays a critical role in aging and in the pathogenesis of several neurological diseases, including ALS, Parkinson’s disease, Alzheimer’s disease, and brain infections, such as bacterial meningitis (94–98). The inflammatory response in the nervous system is one of the primary endogenous sources of ROS in some neurological conditions, and APE1 plays an essential role in these conditions. For example, during neuroinflammation caused by pneumococcal meningitis, higher APE1 expression was observed in the cortex and hippocampus of rats than that in mock-infected animals. Rats supplemented with vitamin B6 showed reduced APE1, glutamate and ROS levels, and decreased cell death and oxidative stress during neuroinflammation (99). Furthermore, in aluminum chloride-induced neuroinflammation in rats, administration of resveratrol as an anti-inflammatory agent was associated with increased APE1 levels and reduced inflammatory responses (100).

The functions of APE1 in inflammatory responses during neuroinflammation are not entirely understood. Some studies have attributed a coactivator role to APE1 redox activity associated with NF-κB and AP-1, promoting proinflammatory cytokines, such as TNFα and IL-8 (10, 18, 52, 101). APE1 translocation from the nucleus to the cytoplasm, followed by p50 reduction, appears to be an essential mechanism for the binding of NF-κB to DNA, thereby triggering inflammation (102, 103). In rats with inflammatory pain, changes in subcellular APE1 distribution can be effected *via* intrathecal injection of E3330, leading to reduced IL-6 levels and alleviation of pain (104). Beyond reducing inflammation, changes in APE1 expression and subcellular distribution also seem to be mediated by APE1 redox function (104).

To observe the role of extranuclear APE1 in regulating neuroinflammatory processes, APE1 with a deleted N-terminal nuclear localization signal (ΔNLS-APE1) was overexpressed in hippocampal astrocytes stimulated with LPS (105). Cytoplasmic APE1 overexpression suppressed NF-κB transcriptional activity and reduced TNFα and iNOS levels, but did not reduce AP-1, showing an anti-inflammatory effect of APE1. These studies also suggested that the inhibitory effect of APE1 on LPS-induced NF-κB activation was not mediated by IκB kinase activity. Additionally, overexpression of APE1 inhibited p300-mediated acetylation of p65 by suppressing intracellular ROS levels following LPS treatment (105). Acetylation of p65 plays a vital role in regulating the inflammatory response (106). The above study demonstrated the involvement of APE1 in this mechanism.

In summary, APE1 plays a multifunctional role in regulating neuroinflammation, acting as an activator or repressor of TFs depending on cellular redox status, APE1 expression level, subcellular compartmentalization, and post-translational modifications, exerting a proinflammatory or anti-inflammatory effect depending on cellular context.

## APE1 in Adaptive Immunity

The adaptive immune system is triggered by responses generated by the innate immune system upon antigen contact at the infection site. T and B-lymphocytes are involved in the adaptive response and responsible for secreting cytokines and antibodies, respectively. These cells can proliferate and differentiate into memory cells with the help of specialized cells in peripheral lymphoid organs, allowing faster and more efficient responses when encountering the same antigen a second time (107, 108).

**Figure 3:**
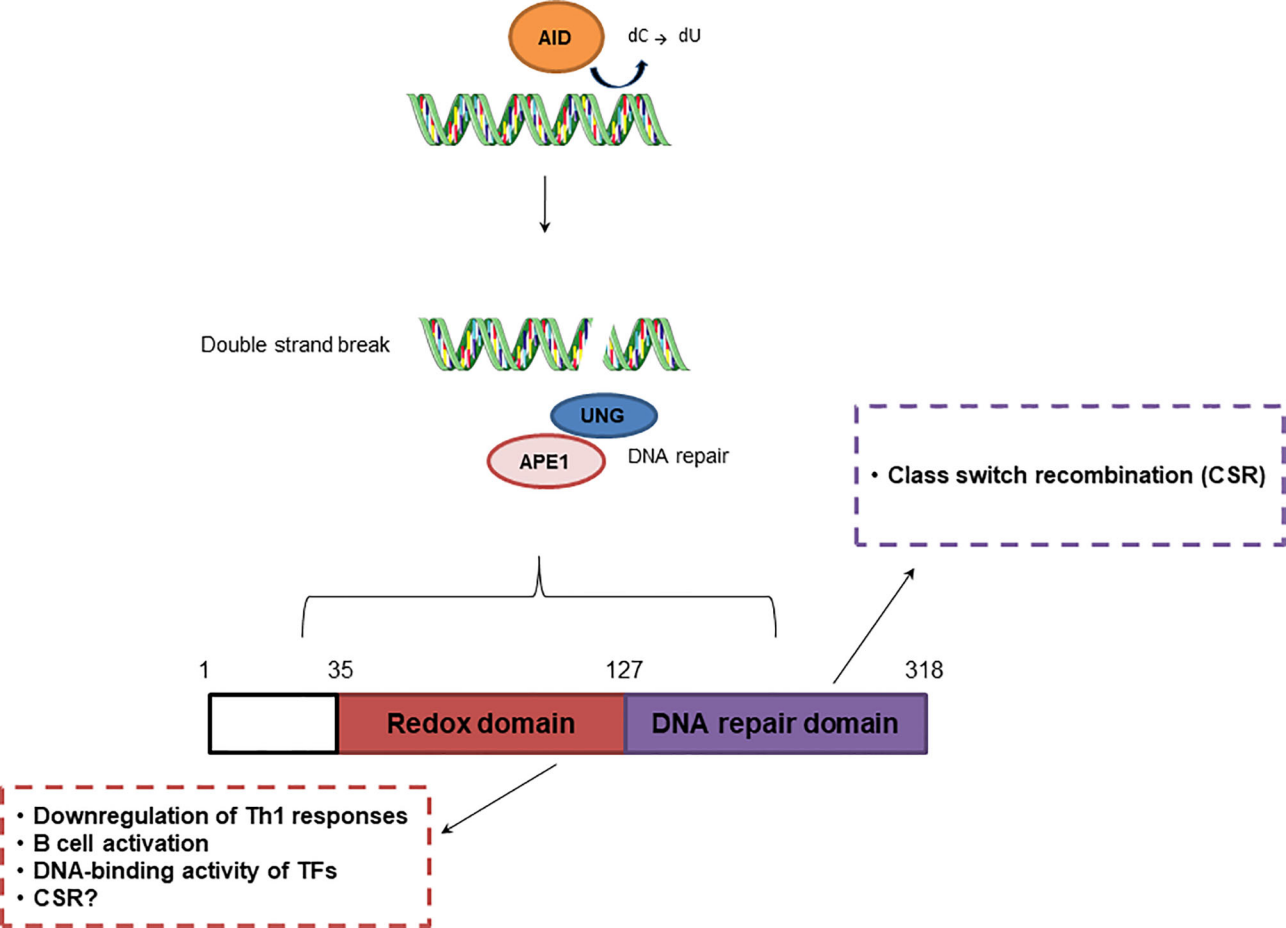
Effect of APE1 redox and repair activities in adaptive immunity. Repair activity of APE1/Ref-1 participates in class switch recombination, while redox activity downregulates Th1 responses and regulates B cell activation.

The role of APE1 in adaptive immunity has been described in several studies (**Figure 3**). According to Akhter et al. (109), the redox activity of APE1 is essential for T helper cell 1 (Th1) response through antigen-presenting cells. The authors observed that in splenocytes from OT-II mice stimulated with ovalbumin, treatment with E3330 increased IFN-γ-producing T cells by altering functions of antigen-presenting cell, suggesting suppression of Th1 immune responses. Inhibition of APE1 redox function induced p38 MAPK activation, upregulation of *IL-12* gene expression, and IL-12 cell surface retention. APE1 redox activity also regulated Pax5a, a TF essential for B cell development. Repression of APE1 protein synthesis blocked CD40-mediated B cell activation by impairing Pax5a activity (110).

Another essential process in the adaptive immune response is class switch recombination (CSR), which is responsible for antibody diversity and is initiated by activation-induced cytidine deaminase (AID) in B cells located in the germinal center (GC) (111). APE1 endonuclease activity is involved in CSR through recognition of AP sites created by uracil-DNA glycosylase (UNG). AID recruits UNG, which converts cytosine into uracil, initiating the BER pathway. This process is crucial for IgA class switching. Although, APE1 redox function regulates CSR, this process is still observed in cells deficient in APE1 endonuclease activity (112). APE1 also mediates CSR through IL-6 signaling, and is involved in proper IgA expression (112, 113). However, once in the GC, B cells undergo somatic hypermutation (SHM), also initiated by AID, and proliferate rapidly, inhibiting the activities of UNG and APE1. APE1 expression is lower in GC B cells than in non-GC B cells and contributes to an increase in SHM (111). According to Xu et al. (114), APE1 endonuclease activity is dispensable for SHM, but may be involved in processing of DNA ends, enabling the ends to participate in CSR.

Due to the multifunctional nature of APE1, researchers have focused on roles of APE1 in tumor cells, and have demonstrated that APE1 expression is associated with poor prognosis in some cancer types including lung, liver, and gastric cancers (115–118). In patients with non-small cell lung cancer, APE1 expression is correlated with tumor-infiltrating lymphocytes, and low APE1 expression together with CD4^+^ T cell infiltration is correlated with good prognosis, suggesting that APE1 levels regulate the function of CD4^+^ T cells (119). APE1 was also found to be active in leukemia T cells; however, when inhibited by E3330, it promoted apoptosis and downregulation of survival genes (120). Furthermore, CD8^+^ T cells and NK cells can release granzymes, such as granzyme A, which cleaves APE1, blocking cellular repair and leading to apoptosis (121). Thus, APE1 could serve as a molecular target for targeted therapies.

Interestingly, APE2 (a less efficient homolog of APE1) was highly expressed in splenic B cells *in vitro*. APE2 deficiency causes severe defects in lymphopoiesis, and prevents B cell progenitors from proliferating in the bone marrow, indicating that APE2 also plays a role in adaptive immunity. APE2 expression is also increased in GC B cells, and protects proliferating B cells from oxidative damage (111, 122–127). Guikema et al. demonstrated that APE1 and APE2 are essential for CSR, as a decrease in this process was observed in splenic B cells of ape1^+/−^, ape2^Y/−^, and double-deficient mice compared to wild type mice (122). However, the role of APE2 in CSR remains unclear, as *APE2* gene deletion in CH12F3 cells does not affect CSR even in APE1 deficient cells (125). APE2 deficient mice show decreased SHM frequency, indicating that APE2 is involved in this process. APE2 interacts with proliferating cell nuclear antigen, facilitating the recruitment of translesion polymerases to AID-induced lesions, which favors an increase in mutagenesis (111, 127). Stavnezer et al. demonstrated that downregulation of APE1 and high expression of APE2 in GC B cells are associated with error-prone repair of AID-induced lesions, and contribute to an increase in mutations in A:T base pairs (111).

## APE1 as a Serologic Biomarker

In recent years, several studies have shown the presence of extracellular APE1 and have suggested its potential use as a biomarker in certain clinical conditions. Although most studies have focused on cancer models which show high APE1 expression (128–131), increasing interest is focused on characterizing APE1 expression in plasma and serum in different diseases, including aging-associated disorders. Serum APE1 levels in patients with coronary artery disease and rheumatoid arthritis have been shown to be elevated compared to levels in healthy controls (132, 133). Furthermore, an experimental murine myocarditis model showed that serum APE1 levels increased until later infection, suggesting the potential use of APE1 as a valuable tool to assess myocardial injury without endomyocardial biopsy (134). Serum APE1 autoantibodies have also been detected in humans. In patients with non-small cell lung cancer, serum APE1 autoantibodies were significantly higher than those in healthy controls, and were closely related to APE1 antigen levels in tumor tissues and peripheral blood (135). Although significant evidence shows that APE1 is delivered through exosomes in response to genotoxic stresses (136), a recent study showed the endogenous hormone 17β-estradiol (E2) significantly increased APE1 secretion in plasma of ovariectomized mice (137). These data suggest that APE1 secretion may also be a natural response in cellular physiology that does not necessarily depend on stress. Therefore, the extracellular functions of APE1 require further investigation.

Some studies have suggested an essential role of APE1 in triggering cell-to-cell communication in the inflammatory response of the local tissue microenvironment. In monocytes secreting APE1 upon inflammatory challenges, extracellular APE1 treatment increased the binding of phospho-p65 to the IL-6 promoter, resulting in activation of gene expression. High IL-6 expression suggests a possible distinct signaling cascade initiated *via* cell surface binding of extracellular APE1 (20). Recently, APE1 was shown to be upregulated in aortic endothelial cells and macrophages of atherosclerotic mice, and its plasma levels were positively correlated with neutrophil/lymphocyte ratios, which indicate systemic inflammation (138). Anti-inflammatory effects have also been associated with extracellular APE1. Using a secretory APE1 adenoviral vector system, Joo et al. evaluated the role of secreted APE1 in cell culture and LPS-induced systemic inflammation in mice. Extracellular APE1 inhibited TNFα-induced VCAM-1 expression in human umbilical vein endothelial cells and LPS-induced Cox-2 expression in Raw264.7 cells. Secreted APE1 in the blood showed an anti-inflammatory effect in mice, as LPS-induced systemic inflammation was reduced together with a decrease in myeloperoxidase release and VCAM-1 expression. This anti-inflammatory effect was associated with APE1 redox function, as mutation in its cysteine residues (C65A/C93A) affected its anti-inflammatory activity. In addition, extracellular APE1 resulted in lower levels of TNFα, IL-1β, and IL-6, and chemotactic cytokines, including MCP-1, in LPS-challenged mice (139). In TNFα-stimulated endothelial cells treated with trichostatin A, an inhibitor of deacetylases, increased protein acetylation, induction of APE1 secretion, and inhibition of TNFα receptor 1, leading to a considerable reduction in VCAM-1 expression were observed. This anti-inflammatory activity may be associated with conformational changes in TNFα receptor 1 *via* thiol-disulfide exchange through the redox activity of extracellular APE1 (140, 141).

To date, the functions of extracellular APE1 and the secretory mechanisms involved are poorly understood. A classical secretory signal peptide is not found in APE1 (141). However, two main nonclassical mechanisms have been proposed: secretion *via* exosomes (136, 142) and ATP-binding cassette (ABC) transporter A1 (143). Furthermore, these signaling mechanisms for APE1 translocation from the nucleus to the cytoplasm and subsequent secretion into the extracellular environment seem to depend on acetylation of lysine residues (K6R/K7R) (141). Acetylation is required to direct APE1 to the plasma membrane for translocation *via* the ABCA1 transporter (143). The importance of APE1 acetylation for modulating DNA repair activity is well known (144), but the reason for acetylation of extracellular APE1 is poorly understood. Cell-to-cell communication in the extracellular environment appears to be insensitive to unmodified extracellular APE1, requiring post-translational modification to trigger responses including cell death. In triple-negative breast cancer cells, acetylated APE1 initiates apoptosis by binding to the receptor for advanced glycation end products, resulting in significant decrease in cell viability (142). Some studies have demonstrated that secreted APE1 retains redox function (140) and DNA repair activity (136). Mangiapane et al. demonstrated that APE1 is secreted through exosomes from several cancer cell lines. The authors identified APE1 p37 and APE1 p33, forms generated by proteasomal degradation, in exosomes. The two forms are enzymatically active, and under genotoxic stress, secretion of APE1 p33 is stimulated, suggesting that APE1 may be a new damage-associated molecular pathway factor, with p33 and p37 forms playing different roles. There is still much to discover about the function of extracellular APE1 and its pathways to establish APE1 as a promising biomarker with high sensitivity and specificity. However, post-translational modifications and complex interactions between APE1 and several targets limit its use as a serological biomarker for specific diseases.

## APE1 Single-Nucleotide Polymorphisms and Immune/Infectious Diseases

Several APE1 variants have been identified in humans (145, 146). Most of these genetic variants are single nucleotide polymorphisms (SNPs) and some have been linked to genomic instability and carcinogenesis (147, 148). Owing to its high frequency in the human population, the most studied and cited *APE1* SNP is rs1130409 (c.444T>A). The nucleotide change (T>A) results in substitution of aspartic acid (D) for glutamic acid (E) at position 148 (D148E), located between the redox and AP endonuclease domains of APE1. Despite its high frequency in the global population (~45%, in dbSNP, NCBI) (149), the clinical significance of this SNP has not been reported in ClinVar, and its functional significance has been predicted to be benign, unknown, or nonexistent (146, 149–151). However, several associations with conditions, including sporadic colorectal, gastric and lung cancers (152–154) and infectious diseases, such as meningitis, have been reported (155). In a study on bacterial meningitis, patients carrying the D148E polymorphism had reduced levels of IL-6, IL-1Ra, IL-8/CXCL8, and MCP-1/CCL2 compared with patients not harboring the polymorphism. In addition, variant allele carriers show more DNA damage accumulation, and children with the D148E allele have a higher IgG/IgA ratio (155). These findings show that this SNP affects the role of APE1 in immunoglobulin production, DNA repair, and expression of cytokines and chemokines. Recently, it was demonstrated that the presence of the D148E polymorphism results in protein structural instability that can affect the ability of APE1 to associate with other BER enzymes (156).

Inflammatory and immune responses are also associated with DNA damage and carcinogenesis. It has been noted that DNA damage and inflammation can promote a positive feedback loop which can drive mutations and consequently, cancer development (157). Immune cells and inflammatory mediators are directly involved in tumor processes, such as angiogenesis, cell proliferation, and invasiveness (158). Meira et al. observed that alkyladenine DNA glycosylase deficiency in a mouse colitis model increased tissue damage and neoplasia development compared to control mice (159). Ulcerative colitis is a chronic inflammatory disease associated with an increased risk of cancer, and Bardia et al. observed that the genotype frequency of *APE1*-D148E was higher in patients with ulcerative colitis than in healthy controls. In addition, they also observed an increase in necrotic and late apoptotic cells and ROS levels in patients harboring this SNP (160). *APE1*-D148E is also associated with the development of colorectal cancer (161). Inhibition of APE1 redox function exerts neuroprotective effects on the enteric nervous system, as observed in a spontaneous chronic colitis mouse model (65).

R237C is another variant associated with endometrial cancer (150) and is characterized by the formation of weaker complexes with DNA and impaired association with downstream enzymes in the BER pathway, including XRCC1 and Pol β. The R237C variant showed an approximately 60% decrease in exonuclease function compared to the wild-type enzyme, and an ~3-fold reduction in 3′ to 5′ exonuclease activity (151) and AP incision capacity in nucleosomes (162), but the AP incision activity on naked DNA was not affected (162).

In a study of patients with immunoglobulin A deficiency and common variable immunodeficiency syndrome, two novel APE1 SNPs were identified: Q51H (rs1048945) and one in the 5′ UTR (rs2307490), only the latter showed an association with common variable immunodeficiency syndrome (163). Another ten polymorphisms were investigated in a study that analyzed the structural effects of amino acid changes in the APE1-DNA complex using predictive methodologies. Two of these were predicted to be deleterious variants, I64T (rs61730854) and P311S (rs1803120), and have been suggested as suitable biomarkers to evaluate the risk of certain diseases (164).

L104R and D283G are uniquely associated with ALS, also known as Lou Gehrig’s disease (165), but this association needs to be confirmed. ALS is a neurodegenerative disease caused by loss of motor neurons and glial reactions. Neuroinflammation is an early event in the development of this disease. Immune system genes *C9orf72*, *TBK1*, and *OPTN* are causative genes for ALS (166). Increased APE1 expression has been observed in patients with ALS (88). Furthermore, increased interaction between APE1 and NPM1, observed in patients with *C9orf72* mutations, suggests high APE1 repair activity (167). However, the roles of APE1 and SNPs in ALS development and their relationships with the immune system require further investigation.

Finally, the influence of APE1 SNPs on redox and repair activities should be further investigated. Furthermore, owing to their varied roles in the immune response, it is necessary to study the effects of APE1 variants on susceptibility to diseases associated with immune, infectious, or inflammatory components.

## APE1 Inhibitors and Potential Therapeutic Drugs

Several compounds have been reported as APE1 inhibitors. Some of these compounds inhibit APE1 directly, while others have indirect actions. Despite the recent discovery of the involvement of AP sites in regulation of inflammatory response, inhibition of DNA repair is not the best alternative for treating inflammatory and immune disorders. Accordingly, inhibitors of endonuclease activity have been investigated for cancer treatment. The overexpression of APE1 is associated with resistance to chemotherapy. Therefore, inhibition of APE1 associated with temozolomide treatment has been used as an alternative to increase chemotherapeutic efficacy in cancer treatment (168).

Methoxyamine (MX) is an alkoxyamine derivative and indirect APE1 endonuclease activity inhibitor. MX can bind to abasic sites, thereby blocking endonuclease activity (169, 170). MX has been studied in clinical trials for the treatment of solid tumors and lymphoma (NCT01851369). Although MX decreased the expression of LPS-induced cytokines and negatively regulated genes involved in prostaglandin production in monocytes (34), the role of MX in inflammatory disorders requires further exploration. Similarly, lucanthone inhibits DNA repair activity of APE1 without affecting the redox function (39) and is in phase II clinical trials for treatment of brain metastases secondary to non-small cell lung cancer (NCT02014545).

APE1 redox function has been studied more in relation with inflammatory and immune disorders due to its role in regulating TFs. The APE1 redox inhibitor E3330 has been suggested as a potential treatment for neoplasms, as it can inhibit the growth and migration of pancreatic tumor cells (63) and also exerts inhibitory effects in other cancer types (13). A recent phase I clinical trial in patients with cancers showed that E3330 treatment was safe (171, 172). However, the therapeutic potential of E3330 in inflammatory diseases requires further exploration. The protective effects of E3330 have been observed in *in vivo* studies of liver diseases, such as alcoholic liver injury (173) and hepatitis in mice, in which E3330 treatment mitigated TNFα, AST, and ALT levels in the plasma (174). In Sprague Dawley rats, E3330 decreases IL-6 expression and inflammatory pain sensitization caused by complete Freund’s adjuvant (104). Recent studies have shown that E3330 could be a promising therapeutic strategy for inflammatory bowel disease. Winnie mice with spontaneous chronic colitis treated with an APE1 inhibitor showed decreased rectal prolapse, edema, and reduced bleeding after 14 days of treatment. In addition, mice also showed decreased loss of mesenteric neurons, reduced oxidative stress, and associated DNA damage (65).

Resveratrol is a natural phenol with antioxidative, anti-inflammatory, anticancer, and anti-neurodegenerative properties (175). *In vitro* studies have shown that resveratrol can inhibit the redox activity of APE1 and decrease AP-1 DNA binding (176). However, it remains unclear whether resveratrol is a direct and specific inhibitor of APE1. In LPS-induced U937 monocytes, the addition of resveratrol did not directly affect APE1 expression, but reduced cytoplasmic localization and acetylation of APE1, contributing to downregulation of the inflammatory response (177). Another natural compound, curcumin, has also been described as an APE1 redox inhibitor. Similar to resveratrol, curcumin exhibits anti-inflammatory, antioxidative, and antineoplastic effects. An *in vitro* study showed that curcumin reduces the APE1 dependent DNA-binding of AP-1 (178). Other studies have shown that curcumin regulates APE1 expression (179, 180). Additionally, isoflavones found in soybeans, including genistein, daidzein, and glycitein, have been studied as APE1 inhibitors and potential therapeutic options for cancer. Isoflavones have been shown to suppress radiation-induced APE1 expression and decrease HIF-1α and NF-κB DNA binding in A549 cells (181). Similar results have been observed in PCa and PC3 cells (182, 183). Liu et al. observed that genistein treatment decreased APE1 expression and TGF-β1, IL-1β, TNFα, and IL-6 levels in the serum of mice with radiation-induced pneumonitis (184). Despite these findings, evidence that these natural compounds act directly to inhibit APE1 remains limited. Therefore, E3330 and its analogs are currently the APE1 inhibitors with the most potential for use in inhibiting the inflammatory response and immune system.

## Conclusions

APE1 plays multiple roles in immune responses, including ROS regulation and cytokine expression in cells mediating innate immunity, including monocytes (34), macrophages (19), keratinocytes (18), dendritic cells (7), neurons (94, 95, 97, 99), and astrocytes (105), and regulation of B cell activation and CSR in adaptive immunity (112). Despite its role in cytokine expression, it is still necessary to determine whether this regulatory control extends to all cell types or is cell type-specific. Additionally, it is necessary to observe how different stimuli influence this regulation. For example, whether the effect of APE1 is the same in a bacterial-triggered response (e.g., LPS), or virus-triggered response, or by transcriptional regulation of inflammatory mediators, also needs more attention.

The recently reported secretion of APE1 in the extracellular environment also plays a role in LPS-induced inflammation (139). However, validation of the use of serum APE1 as a disease biomarker or prognostic marker requires further investigation. Identifying APE1 polymorphisms associated with immune diseases can clarify the full role of APE1 and the consequences of its malfunction in the immune system. Finally, APE1 inhibitors have been extensively studied for cancer treatment, and some studies have also identified their potential effectiveness in inflammatory diseases (174, 178). Thus, APE1 redox inhibitors, such as E3330 may prove to be good alternatives in inflammatory diseases or in controlling inflammation in neoplastic processes.

## Author Contributions

All authors listed have made a substantial, direct, and intellectual contribution to the work, and approved it for publication.

## Funding

This work was supported by the Conselho Nacional de Desenvolvimento Científico e Tecnológico (CNPq-Brazil) and Coordenação de Aperfeiçoamento de Pessoal de Nível Superior (CAPES-Brazil).

## Conflict of Interest

The authors declare that the research was conducted in the absence of any commercial or financial relationships that could be construed as a potential conflict of interest.

## Publisher’s Note

All claims expressed in this article are solely those of the authors and do not necessarily represent those of their affiliated organizations, or those of the publisher, the editors and the reviewers. Any product that may be evaluated in this article, or claim that may be made by its manufacturer, is not guaranteed or endorsed by the publisher.

## References

[1] Whitaker AM Freudenthal BD. APE1: A Skilled Nucleic Acid Surgeon. DNA Repair (Amst) (2018) 71:93–100. doi: 10.1016/j.dnarep.2018.08.012 30170830 PMC6491353

[2] Betlej G Bator E Pyrkosz A Kwiatkowska A. A Dual Face of APE1 in the Maintenance of Genetic Stability in Monocytes: An Overview of the Current Status and Future Perspectives. Genes (Basel) (2020) 11:1–16. doi: 10.3390/genes11060643 PMC7349382 32545201

[3] Whitaker AM Schaich MA Smith MS Flynn TS Freudenthal BD. Base Excision Repair of Oxidative DNA Damage: From Mechanism to Disease. Front Biosci (Landmark Ed (2017) 22:1493–522. doi: 10.2741/4555 PMC5567671 28199214

[4] Hindi NN Elsakrmy N Ramotar D. The Base Excision Repair Process: Comparison Between Higher and Lower Eukaryotes. Cell Mol Life Sci (2021) 78:7943–65. doi: 10.1007/s00018-021-03990-9 PMC11071731 34734296

[5] McNeill DR Whitaker AM Stark WJ Illuzzi JL McKinnon PJ Freudenthal BD. Functions of the Major Abasic Endonuclease (APE1) in Cell Viability and Genotoxin Resistance. Mutagenesis (2020) 35:27. doi: 10.1093/MUTAGE/GEZ046 31816044 PMC7016205

[6] Tell G Quadrifoglio F Tiribelli C Kelley MR. The Many Functions of APE1/Ref-1: Not Only a DNA Repair Enzyme. Antioxid Redox Signal (2009) 11:601–19. doi: 10.1089/ars.2008.2194 PMC2811080 18976116

[7] Cardoso AA Jiang Y Luo M Reed AM Shahda S He Y. APE1/Ref-1 Regulates STAT3 Transcriptional Activity and APE1/Ref-1-STAT3 Dual-Targeting Effectively Inhibits Pancreatic Cancer Cell Survival. PloS One (2012) 7(10):e47462. doi: 10.1371/journal.pone.0047462 23094050 PMC3477158

[8] Logsdon DP Grimard M Luo M Shahda S Jiang Y Tong Y. Regulation of HIF1a Under Hypoxia by APE1/Ref-1 Impacts CA9 Expression: Dual Targeting in Patient-Derived 3D Pancreatic Cancer Models. Mol Cancer Ther (2016) 15:2722–32. doi: 10.1158/1535-7163.MCT-16-0253 PMC5097013 27535970

[9] Fishel ML Jiang Y Rajeshkumar NV Scandura G Anthony L He Y. Impact of APE1/Ref-1 Redox Inhibition on Pancreatic Tumor Growth. Mol Cancer Ther (2011) 10:1698–708. doi: 10.1158/1535-7163.MCT-11-0107.Impact PMC3170439 21700832

[10] Ando K Hirao S Kabe Y Ogura Y Sato I Yamaguchi Y. A New APE1/Ref-1-Dependent Pathway Leading to Reduction of NF-kappaB and AP-1, and Activation of Their DNA-Binding Activity. Nucleic Acids Res (2008) 36:4327–36. doi: 10.1093/nar/gkn416 PMC2490748 18586825

[11] Xanthoudakis S Miao G Wang F Pan YC Curran T. Redox Activation of Fos-Jun DNA Binding Activity is Mediated by a DNA Repair Enzyme. EMBO J (1992) 11:3323–35. doi: 10.1002/j.1460-2075.1992.tb05411.x PMC556867 1380454

[12] Caston RA Gampala S Armstrong L Messmann RA Fishel ML Kelley MR. The Multifunctional APE1 DNA Repair–Redox Signaling Protein as a Drug Target in Human Disease. Drug Discovery Today (2021) 26:218–28. doi: 10.1016/j.drudis.2020.10.015 PMC7855940 33148489

[13] Shah F Logsdon D Messmann RA Fehrenbacher JC Fishel ML Kelley MR. Exploiting the Ref-1-APE1 Node in Cancer Signaling and Other Diseases: From Bench to Clinic. NPJ Precis Oncol (2017) 1:19. doi: 10.1038/s41698-017-0023-0 28825044 PMC5558897

[14] Li M Yang X Lu X Dai N Zhang S Cheng Y. APE1 Deficiency Promotes Cellular Senescence and Premature Aging Features. Nucleic Acids Res (2018) 46:5664–77. doi: 10.1093/nar/gky326 PMC6009672 29750271

[15] Lando D Pongratz I Poellinger L Whitelaw ML. A Redox Mechanism Controls Differential DNA Binding Activities of Hypoxia-Inducible Factor (HIF) 1α and the HIF-Like Factor *. J Biol Chem (2000) 275:4618–27. doi: 10.1074/JBC.275.7.4618 10671489

[16] Huang LE Arany Z Livingston DM Franklin Bunn H. Activation of Hypoxia-Inducible Transcription Factor Depends Primarily Upon Redox-Sensitive Stabilization of its α Subunit. J Biol Chem (1996) 271:32253–9. doi: 10.1074/jbc.271.50.32253 8943284

[17] Nagoya H Futagami S Shimpuku M Tatsuguchi A Wakabayashi T Yamawaki H. Apurinic/apyrimidinic Endonuclease-1 is Associated With Angiogenesis and VEGF Production *via* Upregulation of COX-2 Expression in Esophageal Cancer Tissues. Am J Physiol - Gastrointest Liver Physiol (2014) 306:183–90. doi: 10.1152/ajpgi.00057.2013 PMC5142390 24284961

[18] Lee H-M Yuk J-M Shin D-M Yang C-S Kim K-K Choi D-K. Apurinic/Apyrimidinic Endonuclease 1 Is a Key Modulator of Keratinocyte Inflammatory Responses. J Immunol (2009) 183:6839–48. doi: 10.4049/jimmunol.0901856 19846872

[19] Jedinak A Dudhgaonkar S Kelley MR Sliva D. Apurinic/Apyrimidinic Endonuclease 1 Regulates Inflammatory Response in Macrophages. Anticancer Res (2011) 31:379–85. PMC3256557 21378315

[20] Nath S Roychoudhury S Kling MJ Song H Biswas P Shukla A. The Extracellular Role of DNA Damage Repair Protein APE1 in Regulation of IL-6 Expression. Cell Signal (2017) 39:18–31. doi: 10.1016/j.cellsig.2017.07.019 28751279 PMC5592147

[21] Nassour H Wang Z Saad A Papaluca A Brosseau N Affar EB. Peroxiredoxin 1 Interacts With and Blocks the Redox Factor APE1 From Activating Interleukin-8 Expression. Sci Rep (2016) 6:1–17. doi: 10.1038/srep29389 27388124 PMC4937415

[22] Wang YT Tzeng DW Wang CY Hong JY Yang JL. APE1/Ref-1 Prevents Oxidative Inactivation of ERK for G1-To-S Progression Following Lead Acetate Exposure. Toxicology (2013) 305:120–9. doi: 10.1016/j.tox.2013.01.010 23370007

[23] Guha M Connell MAO Pawlinski R Hollis A Mcgovern P Yan S. Lipopolysaccharide Activation Ofthe MEK-ERK1/2 Pathway in Human Monocytic Cells Mediates Tissue Factor and Tumor Necrosis Factor Expression by Inducing Elk-1 Phosphorylation and Egr-1 Expression. Blood (2009) 98:1429–39. doi: 10.1182/blood.V98.5.1429 11520792

[24] Lindahl T Barnes DE. Repair of Endogenous DNA Damage. Cold Spring Harb Symp Quant Biol (2000) 65:127–33. doi: 10.1101/SQB.2000.65.127 12760027

[25] Allgayer J Kitsera N Bartelt S Epe B Khobta A. Widespread Transcriptional Gene Inactivation Initiated by a Repair Intermediate of 8-Oxoguanine. Nucleic Acids Res (2016) 44:7267–80. doi: 10.1093/nar/gkw473 PMC5009734 27220469

[26] Fleming AM Burrows CJ. 8-Oxo-7,8-Dihydroguanine, Friend and Foe: Epigenetic-Like Regulator Versus Initiator of Mutagenesis. DNA Repair (Amst) (2017) 56:75–83. doi: 10.1016/j.dnarep.2017.06.009 28629775 PMC5548303

[27] Pan L Zhu B Hao W Zeng X Vlahopoulos SA Hazra TK. Oxidized Guanine Base Lesions Function in 8-Oxoguanine DNA Glycosylase-1-Mediated Epigenetic Regulation of Nuclear Factor κb-Driven Gene Expression. J Biol Chem (2016) 291:25553–66. doi: 10.1074/jbc.M116.751453 PMC5207254 27756845

[28] Giorgio M Dellino IG Gambino V Roda N Pelicci PG. On the Epigenetic Role of Guanosine Oxidation. Redox Biol (2020) 29:101398. doi: 10.1016/J.REDOX.2019.101398 31926624 PMC6926346

[29] Ba X. Boldogh Lstvan. 8-Oxoguanine DNA Glycosylase 1: Beyond Repair of the Oxidatively Modified Base Lesions. Redox Biol (2018) 14:669. doi: 10.1016/J.REDOX.2017.11.008 29175754 PMC5975208

[30] Fleming AM Ding Y Burrows CJ. Oxidative DNA Damage Is Epigenetic by Regulating Gene Transcription *via* Base Excision Repair. Proc Natl Acad Sci USA (2017) 114:2604–9. doi: 10.1073/PNAS.1619809114/-/DCSUPPLEMENTAL PMC5347626 28143930

[31] Amente S Bertoni A Morano A Lania L Avvedimento EV Majello B. LSD1-Mediated Demethylation of Histone H3 Lysine 4 Triggers Myc-Induced Transcription. Oncogene (2010) 29:3691. doi: 10.1038/onc.2010.120 20418916

[32] Popov AV Grin IR Dvornikova AP Matkarimov BT Groisman R Saparbaev M. Reading Targeted DNA Damage in the Active Demethylation Pathway: Role of Accessory Domains of Eukaryotic AP Endonucleases and Thymine-DNA Glycosylases. J Mol Biol (2020) 432:1747–68. doi: 10.1016/J.JMB.2019.12.020 31866293

[33] Li M Dai N Wang D Zhong Z. Distinct APE1 Activities Affect the Regulation of VEGF Transcription Under Hypoxic Conditions. Comput Struct Biotechnol J (2019) 17:324–32. doi: 10.1016/j.csbj.2019.02.007 PMC6411614 30906512

[34] Oliveira TT Fontes-Dantas FL de Medeiros Oliveira RK Pinheiro DML Coutinho LG da Silva VL. Chemical Inhibition of Apurinic-Apyrimidinic Endonuclease 1 Redox and DNA Repair Functions Affects the Inflammatory Response *via* Different But Overlapping Mechanisms. Front Cell Dev Biol (2021) 9:731588. doi: 10.3389/FCELL.2021.731588 34616737 PMC8488223

[35] Miller AS Balakrishnan L Buncher NA Opresko PL Bambara RA. Telomere Proteins POT1, TRF1 and TRF2 Augment Long-Patch Base Excision Repair *In Vitro* . Cell Cycle (2012) 11:998–1007. doi: 10.4161/cc.11.5.19483 22336916 PMC3323798

[36] Madlener S Ströbel T Vose S Saydam O Price BD Demple B. Essential Role for Mammalian Apurinic/Apyrimidinic (AP) Endonuclease Ape1/Ref-1 in Telomere Maintenance. PNAS (2013) 110:17844–9. doi: 10.1073/pnas.1304784110 PMC3816401 24127576

[37] Malfatti MC Antoniali G Codrich M Tell G. Coping With RNA Damage With a Focus on APE1, a BER Enzyme at the Crossroad Between DNA Damage Repair and RNA Processing/Decay. DNA Repair (Amst) (2021) 104:103133. doi: 10.1016/j.dnarep.2021.103133 34049077

[38] Kim WC King D Lee CH. RNA-Cleaving Properties of Human Apurinic/Apyrimidinic Endonuclease 1 (APE1). Int J Biochem Mol Biol (2010) 1:12. 21968700 PMC3180037

[39] Luo M Kelley MR. Inhibition of the Human Apurinic/Apyrimidinic Endonuclease (Ape1) Repair Activity and Sensitization of Breast Cancer Cells to DNA Alkylating Agents With Lucanthone. Anticancer Res (2004) 24:2127–34. 15330152

[40] Li P Chang M. Roles of PRR-Mediated Signaling Pathways in the Regulation of Oxidative Stress and Inflammatory Diseases. Int J Mol Sci (2021) 22(14):1–20. doi: 10.3390/IJMS22147688 PMC8306625 34299310

[41] Newton K Dixit VM. Signaling in Innate Immunity and Inflammation. Cold Spring Harb Perspect Biol (2012) 4(3):1–19. doi: 10.1101/CSHPERSPECT.A006049 PMC3282411 22296764

[42] Mittal M Siddiqui MR Tran K Reddy SP Malik AB. Reactive Oxygen Species in Inflammation and Tissue Injury. Antioxidants Redox Signal (2014) 20:1126–67. doi: 10.1089/ars.2012.5149 PMC3929010 23991888

[43] Ragu S Matos-Rodrigues G Lopez BS. Replication Stress, DNA Damage, Inflammatory Cytokines and Innate Immune Response. Genes (Basel) (2020) 11(4):1–26. doi: 10.3390/genes11040409 PMC7230342 32283785

[44] Tian T Wang Z Zhang J. Pathomechanisms of Oxidative Stress in Inflammatory Bowel Disease and Potential Antioxidant Therapies. Oxid Med Cell Longev (2017) 2017:1–18. doi: 10.1155/2017/4535194 PMC5506473 28744337

[45] Aviello G Knaus UG. NADPH Oxidases and ROS Signaling in the Gastrointestinal Tract. Mucosal Immunol (2018) 11:1011–23. doi: 10.1038/s41385-018-0021-8 29743611

[46] Paracha UZ Fatima K Alqahtani M Chaudhary A Abuzenadah A Damanhouri G. Oxidative Stress and Hepatitis C Virus. Virol J (2013) 10:1–9. doi: 10.1186/1743-422X-10-251 23923986 PMC3751576

[47] Kattoor AJ Pothineni NVK Palagiri D Mehta JL. Oxidative Stress in Atherosclerosis. Curr Atheroscler Rep (2017) 19:1–11. doi: 10.1007/s11883-017-0678-6 28921056

[48] Ohl K Tenbrock K Kipp M. Oxidative Stress in Multiple Sclerosis: Central and Peripheral Mode of Action. Exp Neurol (2016) 277:58–67. doi: 10.1016/j.expneurol.2015.11.010 26626971 PMC7094520

[49] Flaherty DM Monick MM Carter AB Peterson MW Hunninghake GW. Oxidant-Mediated Increases in Redox Factor-1 Nuclear Protein and Activator Protein-1 DNA Binding in Asbestos-Treated Macrophages. J Immunol (2002) 168(11):5675–81. doi: 10.4049/jimmunol.168.11.5675 12023366

[50] Hsieh MM Hegde V Kelley MR Deutsch WA. Activation of APE/Ref-1 Redox Activity is Mediated by Reactive Oxygen Species and PKC Phosphorylation. Nucleic Acids Res (2001) 29:3116–22. doi: 10.1093/NAR/29.14.3116 PMC55809 11452037

[51] Ding SZ O’Hara AM Denning TL Dirden-Kramer B Mifflin RC Reyes VE. Helicobacter Pylori and H2O2 Increase AP Endonuclease-1/Redox Factor-1 Expression in Human Gastric Epithelial Cells. Gastroenterology (2004) 127:845–58. doi: 10.1053/j.gastro.2004.06.017 15362040

[52] O’Hara AM Bhattacharyya A Mifflin RC Smith MF Ryan KA Scott KG-E. Interleukin-8 Induction by Helicobacter Pylori in Gastric Epithelial Cells is Dependent on Apurinic/Apyrimidinic Endonuclease-1/Redox Factor-1. J Immunol (2006) 177:7990–9. doi: 10.4049/jimmunol.177.11.7990 17114472

[53] O’Hara AM Bhattacharyya A Bai J Mifflin RC Ernst PB Mitra S. Tumor Necrosis Factor (TNF)-α-Induced IL-8 Expression in Gastric Epithelial Cells: Role of Reactive Oxygen Species and AP Endonuclease-1/Redox Factor (Ref)-1. Cytokine (2009) 46:359–69. doi: 10.1016/j.cyto.2009.03.010 PMC2846768 19376732

[54] Frossi B De Carli M Daniel KC Rivera J Pucillo C. Oxidative Stress Stimulates IL-4 and IL-6 Production in Mast Cells by an APE/Ref-1-Dependent Pathway. Eur J Immunol (2003) 33:2168–77. doi: 10.1002/eji.200323995 12884291

[55] den Hartog G Chattopadhyay R Ablack A Hall EH Butcher LD Bhattacharyya A. Regulation of Rac1 and Reactive Oxygen Species Production in Response to Infection of Gastrointestinal Epithelia. PloS Pathog (2016) 12:1–20. doi: 10.1371/journal.ppat.1005382 PMC4711900 26761793

[56] Zhao T Zhang H Guo Y Fan Z. Granzyme K Directly Processes Bid to Release Cytochrome C and Endonuclease G Leading to Mitochondria-Dependent Cell Death. J Biol Chem (2007) 282(16):12104–11. doi: 10.1074/jbc.M611006200 17308307

[57] Guo Y Chen J Zhao T Fan Z. Granzyme K Degrades the Redox/DNA Repair Enzyme Ape1 to Trigger Oxidative Stress of Target Cells Leading to Cytotoxicity. Mol Immunol (2008) 45:2225–35. doi: 10.1016/j.molimm.2007.11.020 18179823

[58] Mitomo K Nakayamaa K Fujimotoa K Suna X Sekib S Yamamotoa K. Two Different Cellular Redox Systems Regulate the DNA-Binding Activity of the P50 Subunit of NF-κb *In Vitro* . Gene (1994) 145:197–203. doi: 10.1016/0378-1119(94)90005-1 8056331

[59] Cesaratto L Codarin E Vascotto C Leonardi A Kelley MR Tiribelli C. Specific Inhibition of the Redox Activity of Ape1/Ref-1 by E3330 Blocks Tnf-A-Induced Activation of Il-8 Production in Liver Cancer Cell Lines. PloS One (2013) 8(8):1–13. doi: 10.1371/journal.pone.0070909 PMC3744539 23967134

[60] Miyamoto K Nagakawa J Hishinuma I Hirota K Yasuda M Yamanaka T. Suppressive Effects of E3330, a Novel Quinone Derivative, on Tumor Necrosis Factor-α Generation From Monocytes and Macrophages. Agents Actions (1992) 37:297–304. doi: 10.1007/BF02028123 1284192

[61] Su D Delaplane S Luo M Rempel DL Vu B Kelley MR. Interactions of Apurinic/apyrimidinic Endonuclease With a Redox Inhibitor: Evidence for an Alternate Conformation of the Enzyme. Biochemistry (2011) 50:82–92. doi: 10.1021/bi101248s 21117647 PMC3070192

[62] Zhang J Luo M Marasco D Logsdon D Lafavers KA Chen Q. Inhibition of Apurinic/Apyrimidinic Endonuclease I’s Redox Activity Revisited. Biochemistry (2013) 52:2955–66. doi: 10.1021/bi400179m PMC3706204 23597102

[63] Zou G Maitra A. Small-Molecule Inhibitor of the AP Endonuclease 1 / REF-1 E3330 Inhibits Pancreatic Cancer Cell Growth and Migration. Mol Cancer Ther (2008) 7(7):2012–22. doi: 10.1158/1535-7163.MCT-08-0113 PMC3569736 18645011

[64] Kelley MR Luo M Reed A Su D Delaplane S Borch RF. Functional Analysis of Novel Analogues of E3330 That Block the Redox Signaling Activity of the Multifunctional AP Endonuclease/Redox Signaling Enzyme APE1/Ref-1. Antioxid Redox Signal (2011) 14:1387–401. doi: 10.1089/ARS.2010.3410 PMC3061197 20874257

[65] Sahakian L Filippone RT Stavely R Robinson AM Yan XS Abalo R. Inhibition of APE1/Ref-1 Redox Signaling Alleviates Intestinal Dysfunction and Damage to Myenteric Neurons in a Mouse Model of Spontaneous Chronic Colitis. Inflammation Bowel Dis (2021) 27:388–406. doi: 10.1093/ibd/izaa161 PMC8287929 32618996

[66] Hu Z Hui B Hou X Liu R Sukhanov S Liu X. APE1 Inhibits Foam Cell Formation From Macrophages *via* LOX1 Suppression. Am J Transl Res (2020) 12:6559. 33194052 PMC7653594

[67] Yuk JM Yang CS Shin DM Kim KK Lee SK Song YJ. A Dual Regulatory Role of Apurinic/Apyrimidinic Endonuclease 1/Redox Factor-1 in HMGB1-Induced Inflammatory Responses. Antioxidants Redox Signal (2009) 11:575–88. doi: 10.1089/ars.2008.2196 18715145

[68] Maruyama K Nakaga N Aonuma T Saito Y Hayasaka T. The Antioxidant and DNA-Repair Enzyme Apurinic / Apyrimidinic Endonuclease 1 Limits the Development of Tubulointerstitial Fibrosis Partly by Modulating the Immune System. Sci Rep (2019) 9:1–11. doi: 10.1038/s41598-019-44241-z 31127150 PMC6534557

[69] Wunderlich R Ruehle PF Deloch L Unger K Hess J Zitzelsberger H. Interconnection Between DNA Damage, Senescence, Inflammation, and Cancer. Front Biosci - Landmark (2017) 22:348–69. doi: 10.2741/4488 27814618

[70] Cramer T Yamanishi Y Clausen BE Förster I Pawlinski R Mackman N. HIF-1α is Essential for Myeloid Cell-Mediated Inflammation. Cell (2003) 112:645–57. doi: 10.1016/S0092-8674(03)00154-5 PMC4480774 12628185

[71] Yu H Pardoll D Jove R. STATs in Cancer Inflammation and Immunity: A Leading Role for STAT3. Nat Rev Cancer (2009) 9:798–809. doi: 10.1038/NRC2734 19851315 PMC4856025

[72] Hillmer EJ Zhang H Li HS Watowich SS. STAT3 Signaling in Immunity. Cytokine Growth Factor Rev (2016) 31:1–15. doi: 10.1016/j.cytogfr.2016.05.001 27185365 PMC5050093

[73] Cummins EP Keogh CE Crean D Taylor CT. The Role of HIF in Immunity and Inflammation. Mol Aspects Med (2016) 47–48:24–34. doi: 10.1016/j.mam.2015.12.004 26768963

[74] McMahon SB Monroe JG. The Role of Early Growth Response Gene 1 (Egr-1) in Regulation of the Immune Response. J Leukoc Biol (1996) 60:159–66. doi: 10.1002/jlb.60.2.159 8773576

[75] Huang RP Adamson ED. Characterization of the DNA-Binding Properties of the Early Growth Response-1 (Egr-1) Transcription Factor: Evidence for Modulation by a Redox Mechanism. DNA Cell Biol (1993) 12:265–73. doi: 10.1089/dna.1993.12.265 8466649

[76] Muñoz-Fontela C Mandinova A Aaronson SA Lee SW. Emerging Roles of P53 and Other Tumour-Suppressor Genes in Immune Regulation. Nat Rev Immunol (2016) 16:741–50. doi: 10.1038/nri.2016.99 PMC5325695 27667712

[77] Jayaraman L Murthy KGK Zhu C Curran T Xanthoudakis S Prives C. Identification of Redox/Repair Protein Ref-1 as a Potent Activator of P53. Genes Dev (1997) 11:558–70. doi: 10.1101/gad.11.5.558 9119221

[78] Codrich M Comelli M Malfatti MC Mio C Ayyildiz D. Inhibition of APE1-Endonuclease Activity Affects Cell Metabolism in Colon Cancer Cells *via* a P53-Dependent Pathway. DNA Repair (Amst) (2020) 82:1–36. doi: 10.1016/j.dnarep.2019.102675.Inhibition PMC7092503 31450087

[79] Cobaleda C Schebesta A Delogu A Busslinger M. Pax5: The Guardian of B Cell Identity and Function. Nat Immunol (2007) 8:463–70. doi: 10.1038/ni1454 17440452

[80] Tell G Zecca A Pellizzari L Spessotto P Colombatti A Kelley MR. An “Environment to Nucleus” Signaling System Operates in B Lymphocytes: Redox Status Modulates BSAP/Pax-5 Activation Through Ref-1 Nuclear Translocation. Nucleic Acids Res (2000) 28:1099–105. doi: 10.1093/nar/28.5.1099 PMC102597 10666449

[81] Fantini D Fantini D Vascotto C Deganuto M Bivi N Gustincich S. APE1/Ref-1 Regulates PTEN Expression Mediated by Egr-1. Free Radic Res (2008) 42:20–9. doi: 10.1080/10715760701765616 PMC2677450 18324520

[82] Lehmann U Brocke P Dittmer J Nordheim A. Characterization of the Human Elk-1 Promoter. Potential Role of a Downstream Intronic Sequence for Elk-1 Gene Expression in Monocytes. J Biol Chem (1999) 274:1736–44. doi: 10.1074/jbc.274.3.1736 9880555

[83] Qu K Ma Xf Li Gh Zhang H Liu Y Zhang K. Vitamin C Down-Regulate Apo(a) Expression *via* Tet2-Dependent DNA Demethylation in HepG2 Cells. Int J Biol Macromol (2017) 98:637–45. doi: 10.1016/j.ijbiomac.2017.02.025 28192139

[84] Guha M Mackman N. LPS Induction of Gene Expression in Human Monocytes. Cell Signal (2001) 13:85–94. doi: 10.1016/S0898-6568(00)00149-2 11257452

[85] Dragunow M. Ref-1 Expression in Adult Mammalian Neurons and Astrocytes. Neumsci Lett (1995) 191:189–92. doi: 10.1016/0304-3940(95)11589-o 7644143

[86] Tan Y Nakagawa Y Akiyama K Wakabayashi H Sarker AH Seki S. cDNA Cloning of Rat Major AP Endonuclease (APEX Nuclease) and Analyses of Its mRNA Expression in Rat Tissues. Acta Med Okayama (1996) 50(1):53–60. doi: 10.18926/AMO/30516 8701782

[87] Ono Y Watanabe M Inoue Y Ohmoto T Akiyama K Tsutsui K. Developmental Expression of APEX Nuclease, a Multifunctional DNA Repair Enzyme, in Mouse Brains. Dev Brain Res (1995) 86:1–6. doi: 10.1016/0165-3806(94)00212-I 7656403

[88] Shaikh AY Martin LJ. DNA Base-Excision Repair Enzyme Apurinic/Apyrimidinic Endonuclease/Redox Factor-1 is Increased and Competent in the Brain and Spinal Cord of Individuals With Amyotrophic Lateral Sclerosis. NeuroMol Med (2002) 2:47–60. doi: 10.1385/NMM:2:1:47 12230304

[89] Domenis R Bergamin N Gianfranceschi G Vascotto C Romanello M Rigo S. The Redox Function of APE1 is Involved in the Differentiation Process of Stem Cells Toward a Neuronal Cell Fate. PloS One (2014) 9:e89232. doi: 10.1371/journal.pone.0089232 24586617 PMC3929656

[90] Stetler RA Gao Y Zukin RS Vosler PS Zhang L Zhang F. Apurinic/apyrimidinic Endonuclease APE1 is Required for PACAP-Induced Neuroprotection Against Global Cerebral Ischemia. Proc Natl Acad Sci USA (2010) 107(7):3204–9. doi: 10.1073/pnas.1000030107 PMC2840276 20133634

[91] Leak RK Li P Zhang F Sulaiman HH Weng Z Wang G. Apurinic/apyrimidinic Endonuclease 1 Upregulation Reduces Oxidative DNA Damage and Protects Hippocampal Neurons From Ischemic Injury. Antioxidants Redox Signal (2015) 22:135–48. doi: 10.1089/ars.2013.5511 PMC4281843 24180454

[92] Yang J-L Chen W-Y Mukda S Yang Y-R Sun S-F Chen S-D. Oxidative DNA Damage is Concurrently Repaired by Base Excision Repair (BER) and Apyrimidinic Endonuclease 1 (APE1)-Initiated Nonhomologous End Joining (NHEJ) in Cortical Neurons. Neuropathol Appl Neurobiol (2020) 46:375–90. doi: 10.1111/nan.12584 PMC7317839 31628877

[93] Kim HS Guo C Thompson EL Jiang Y Kelley MR Vasko MR. APE1, the DNA Base Excision Repair Protein, Regulates the Removal of Platinum Adducts in Sensory Neuronal Cultures by NER. Mutat Res Mol Mech Mutagen (2015) 779:96–104. doi: 10.1016/J.MRFMMM.2015.06.010 PMC4554977 26164266

[94] Liguori I Russo G Curcio F Bulli G Aran L Della-Morte D. Oxidative Stress, Aging, and Diseases. Clin Interv Aging (2018) 13:757–72. doi: 10.2147/CIA.S158513 PMC5927356 29731617

[95] Bond L Bernhardt K Madria P Sorrentino K Scelsi H Mitchell CS. A Metadata Analysis of Oxidative Stress Etiology in Preclinical Amyotrophic Lateral Sclerosis: Benefits of Antioxidant Therapy. Front Neurosci (2018) 12:10. doi: 10.3389/fnins.2018.00010 29416499 PMC5787557

[96] Wei Z Li X Li X Liu Q Cheng Y. Oxidative Stress in Parkinson’s Disease: A Systematic Review and Meta-Analysis. Front Mol Neurosci (2018) 11:236. doi: 10.3389/fnmol.2018.00236 30026688 PMC6041404

[97] Tobore TO. On the Central Role of Mitochondria Dysfunction and Oxidative Stress in Alzheimer’s Disease. Neurol Sci (2019) 40(8):1527–40. doi: 10.1007/s10072-019-03863-x 30982132

[98] Klein M Koedel U Pfister HW. Oxidative Stress in Pneumococcal Meningitis: A Future Target for Adjunctive Therapy? Prog Neurobiol (2006) 80:269–80. doi: 10.1016/J.PNEUROBIO.2006.11.008 17215069

[99] Coutinho LG de Oliveira AHS Witwer M Leib SL Agnez-Lima LF. DNA Repair Protein APE1 Is Involved in Host Response During Pneumococcal Meningitis and Its Expression Can Be Modulated by Vitamin B6. J Neuroinflamm (2017) 14:243. doi: 10.1186/s12974-017-1020-5 PMC5727666 29233148

[100] Zaky A Mohammad B Moftah M Kandeel KM Bassiouny AR. Apurinic/apyrimidinic Endonuclease 1 is a Key Modulator of Aluminum-Induced Neuroinflammation. BMC Neurosci (2013) 14:1–12. doi: 10.1186/1471-2202-14-26 23497276 PMC3616857

[101] Guan Z Basi D Li Q Mariash A Xia YF Geng JG. Loss of Redox Factor 1 Decreases NF-κb Activity and Increases Susceptibility of Endothelial Cells to Apoptosis. Arterioscler Thromb Vasc Biol (2005) 25:96–101. doi: 10.1161/01.ATV.0000150418.14698.75 15539619

[102] Katsuyuki M Kohzo N Kotaro F Xiangao S Shuji S Ken-ichi Y. Two Different Cellular Redox Systems Regulate the DNA-Binding Activity of the P50 Subunit of NF-Kappa B *In Vitro* . Gene (1994) 145:197–203. doi: 10.1016/0378-1119(94)90005-1 8056331

[103] Tell G Damante G Caldwell D Kelley MR. The Intracellular Localization of APE1/Ref-1: More Than a Passive Phenomenon? Antioxid Redox Signal (2005) 7:367–84. doi: 10.1089/ARS.2005.7.367 15706084

[104] Zaky A Bouali-Benazzouz R Favereaux A Tell G Landry M. APE1/Ref-1 Redox Function Contributes to Inflammatory Pain Sensitization. Exp Neurol (2018) 307:1–11. doi: 10.1016/j.expneurol.2018.05.014 29772245

[105] Baek H Lim CS Byun HS Cho HS Lee YR Shin YS. The Anti-Inflammatory Role of Extranuclear Apurinic/Apyrimidinic Endonuclease 1/Redox Effector Factor-1 in Reactive Astrocytes. Mol Brain (2016) 9:1–12. doi: 10.1186/s13041-016-0280-9 27986089 PMC5162091

[106] Ishinaga H Jono H Lim JH Kweon S-M Xu H Ha U-H. TGF-Beta Induces P65 Acetylation to Enhance Bacteria-Induced NF-kappaB Activation. EMBO J (2007) 26:1150–62. doi: 10.1038/sj.emboj.7601546 PMC1852843 17268554

[107] Keim C Kazadi D Rothschild G Basu U. Regulation of AID, the B-Cell Genome Mutator. Genes Dev (2013) 27(1):1–17. doi: 10.1101/gad.200014.112 23307864 PMC3553278

[108] Noakes PS Michaelis LJ. Innate and Adaptive Immunity. In: Diet, Immunity and Inflammation. Cambridge: Woodhead Publishing. p. 3–33. doi: 10.1533/9780857095749.1.3

[109] Akhter N Takeda Y Nara H Araki A Ishii N Asao N. Apurinic/apyrimidinic Endonuclease 1/Redox Factor-1 (Ape1/Ref-1) Modulates Antigen Presenting Cell-Mediated T Helper Cell Type 1 Responses. J Biol Chem (2016) 291(45):23672–80. doi: 10.1074/jbc.M116.742353 PMC5095420 27637330

[110] Merluzzi S Moretti M Altamura S Zwollo P Sigvardsson M Vitale G. CD40 Stimulation Induces Pax5/BSAP and EBF Activation Through a APE/Ref-1-Dependent Redox Mechanism *. J Biol Chem (2004) 279:1777–86. doi: 10.1074/JBC.M305418200 14594818

[111] Stavnezer J Linehan EK Thompson MR Habboub G Ucher AJ Kadungure T. Differential Expression of APE1 and APE2 in Germinal Centers Promotes Error-Prone Repair and A:T Mutations During Somatic Hypermutation. Proc Natl Acad Sci USA (2014) 111(25):9217–22. doi: 10.1073/pnas.1405590111 PMC4078814 24927551

[112] Frossi B Antoniali G Yu XK Akhtar N Kaplan XMH Kelley MR. Endonuclease and Redox Activities of Human Apurinic / Apyrimidinic Endonuclease 1 Have Distinctive and Essential Functions in IgA Class Switch Recombination. J Biol Chem (2019) 294:5198–207. doi: 10.1074/jbc.RA118.006601 PMC6442068 30705092

[113] Amirifar P Yazdani R Azizi G Ranjouri MR Durandy A Plebani A. Known and Potential Molecules Associated With Altered B Cell Development Leading to Predominantly Antibody Deficiencies. Pediatr Allergy Immunol (2021) 32:1601–15. doi: 10.1111/pai.13589 34181780

[114] Xu J Husain A Hu W Honjo T Kobayashi M. Ape1 is Dispensable for s-Region Cleavage But Required for Its Repair in Class Switch Recombination. Proc Natl Acad Sci USA (2014) 111(48):17242–7. doi: 10.1073/pnas.1420221111 PMC4260585 25404348

[115] Wang D Xiang DB Yang Xq Chen LS Li MX Zhong ZY. APE1 Overexpression is Associated With Cisplatin Resistance in non-Small Cell Lung Cancer and Targeted Inhibition of APE1 Enhances the Activity of Cisplatin in A549 Cells. Lung Cancer (2009) 66. doi: 10.1016/j.lungcan.2009.02.019 19324449

[116] Li M Wilson DM. Human Apurinic/Apyrimidinic Endonuclease 1. Antioxidants Redox Signal (2014) 20(4):678–707. doi: 10.1089/ars.2013.5492 PMC3901322 23834463

[117] Kan G Dong W. The Expression of PD-L1 APE1 and P53 in Hepatocellular Carcinoma and its Relationship to Clinical Pathology. Eur Rev Med Pharmacol Sci (2015) 19:3063–71. 26367730

[118] Qing Y Li Q Ren T Xia W Peng Y Liu GL. Upregulation of PD-L1 and APE1 is Associated With Tumorigenesis and Poor Prognosis of Gastric Cancer. Drug Des Devel Ther (2015) 9:901–9. doi: 10.2147/DDDT.S75152 PMC4338255 25733810

[119] Li Y Zhao X Xiao H Yang B Liu J Rao W. APE1 may Influence CD4+ Naïve T Cells on Recurrence Free Survival in Early Stage NSCLC. BMC Cancer (2021) 21:1–11. doi: 10.1186/s12885-021-07950-1 33676448 PMC7937314

[120] Ding J Fishel ML Reed AM McAdams E Czader MB Cardoso AA. Ref-1/APE1 as a Transcriptional Regulator and Novel Therapeutic Target in Pediatric T-Cell Leukemia. Mol Cancer Ther (2017) 16:1401–11. doi: 10.1158/1535-7163.MCT-17-0099 PMC5500420 28446640

[121] Fan Z Beresford PJ Zhang D Xu Z Novina CD Yoshida A. Cleaving the Oxidative Repair Protein Ape I Enhances Cell Death Mediated by Granzyme A. Nat Immunol (2003) 4:145–53. doi: 10.1038/ni885 12524539

[122] Guikema JEJ Linehan EK Tsuchimoto D Nakabeppu Y Strauss PR Stavnezer J. APE1- And APE2-Dependent DNA Breaks in Immunoglobulin Class Switch Recombination. J Exp Med (2007) 204:3017–26. doi: 10.1084/jem.20071289 PMC2118529 18025127

[123] Ide Y Tsuchimoto D Tominaga Y Nakashima M Watanabe T Sakumi K. Growth Retardation and Dyslymphopoiesis Accompanied by G2/M Arrest in APEX2-Null Mice. Blood (2004) 104(13):4097–103. doi: 10.1182/blood-2004-04-1476 15319281

[124] Guikema JEJ Gerstein RM Linehan EK Cloherty EK Evan-Browning E Tsuchimoto D. Apurinic/Apyrimidinic Endonuclease 2 Is Necessary for Normal B Cell Development and Recovery of Lymphoid Progenitors After Chemotherapeutic Challenge. J Immunol (2011) 186(4):1943–50. doi: 10.4049/jimmunol.1002422 PMC4041036 21228350

[125] Masani S Han L Yu K. Apurinic/apyrimidinic Endonuclease 1 is the Essential Nuclease During Immunoglobulin Class Switch Recombination. Mol Cell Biol (2013) 33:1468–73. doi: 10.1128/MCB.00026-13 PMC3624277 23382073

[126] Chaudhari S Ware AP Jayaram P Gorthi SP El-Khamisy SF Satyamoorthy K. Apurinic/Apyrimidinic Endonuclease 2 (APE2): An Ancillary Enzyme for Contextual Base Excision Repair Mechanisms to Preserve Genome Stability. Biochimie (2021) 190:70–90. doi: 10.1016/J.BIOCHI.2021.07.006 34302888

[127] Guikema JEJ Linehan EK Esa N Tsuchimoto D Nakabeppu Y Woodland RT. Apurinic/Apyrimidinic Endonuclease 2 Regulates the Expansion of Germinal Centers by Protecting Against Activation-Induced Cytidine Deaminase–Independent DNA Damage in B Cells. J Immunol (2014) 193:931–9. doi: 10.4049/jimmunol.1400002 PMC4105697 24935922

[128] Shin JH Choi S Lee YR Park MS Na YG Irani K. APE1/ref-1 as a Serological Biomarker for the Detection of Bladder Cancer. Cancer Res Treat (2015) 47:823–33. doi: 10.4143/crt.2014.074 PMC4614188 25672588

[129] Pascut D Sukowati CHC Antoniali G Mangiapane G Burra S Mascaretti LG. Serum AP-Endonuclease 1 (Sape1) as Novel Biomarker for Hepatocellular Carcinoma. Oncotarget (2019) 10:383. doi: 10.18632/ONCOTARGET.26555 30719231 PMC6349448

[130] Lee YR Park MS Joo HK Kim KM Kim J Jeon BH. Therapeutic Positioning of Secretory Acetylated APE1/Ref-1 Requirement for Suppression of Tumor Growth in Triple-Negative Breast Cancer *In Vivo* . Sci Rep (2018) 8:1–16. doi: 10.1038/s41598-018-27025-9 29880821 PMC5992149

[131] Lee YR Joo HK Jeon BH. The Biological Role of Apurinic/Apyrimidinic Endonuclease1/Redox Factor-1 as a Therapeutic Target for Vascular Inflammation and as a Serologic Biomarker. Biomedicines (2020) 8(3):1–12. doi: 10.3390/BIOMEDICINES8030057 PMC7148461 32164272

[132] Jin SA Seo HJ Kim SK Lee YR Choi S Ahn KT. Elevation of the Serum Apurinic/Apyrimidinic Endonuclease 1/Redox Factor-1 in Coronary Artery Disease. Korean Circ J (2015) 45:364–71. doi: 10.4070/kcj.2015.45.5.364 PMC4580694 26413103

[133] Yoo IS Lee Y Kang SW Kim J Joo H Yoo S. Elevated APE1 / Ref-1 Levels of Synovial Fluids in Patients With Rheumatoid Arthritis : Reflection of Disease Activity. J Clin Med (2021) 10:1–11. doi: 10.3390/jcm10225324 PMC8621376 34830606

[134] Jin SA Lim BK Seo HJ Kim SK Ahn KT Jeon BH. Elevation of Serum APE1/Ref-1 in Experimental Murine Myocarditis. Int J Mol Sci (2017) 18:1–8. doi: 10.3390/ijms18122664 PMC5751266 29292734

[135] Dai N Cao XJ Li MX Qing Y Liao L Lu XF. Serum APE1 Autoantibodies: A Novel Potential Tumor Marker and Predictor of Chemotherapeutic Efficacy in Non-Small Cell Lung Cancer. PloS One (2013) 8:1–9. doi: 10.1371/journal.pone.0058001 PMC3589448 23472128

[136] Mangiapane G Parolini I Conte K Malfatti MC Corsi J Sanchez M. Enzymatically Active Apurinic/Apyrimidinic Endodeoxyribonuclease 1 Is Released by Mammalian Cells Through Exosomes. J Biol Chem (2021) 296:1–16. doi: 10.1016/j.jbc.2021.100569 PMC8080531 33753167

[137] Lee Y-R Joo H-K Lee E-O Kim S Jin H Choi Y-H. 17β-Estradiol Increases APE1/Ref-1 Secretion in Vascular Endothelial Cells and Ovariectomized Mice: Involvement of Calcium-Dependent Exosome Pathway. Biomedicines (2021) 9(8):1–16. doi: 10.3390/biomedicines9081040 PMC8394342 34440244

[138] Lee YR Joo HK Lee EO Park MS Cho HS Kim S. Plasma APE1/Ref-1 Correlates With Atherosclerotic Inflammation in ApoE-/- Mice. Biomedicines (2020) 8(9):1–16. doi: 10.3390/BIOMEDICINES8090366 PMC7555038 32967121

[139] Joo HK Lee YR Lee EO Park MS Choi S Kim CS. The Extracellular Role of Ref-1 as Anti-Inflammatory Function in Lipopolysaccharide-Induced Septic Mice. Free Radic Biol Med (2019) 139:16–23. doi: 10.1016/j.freeradbiomed.2019.05.013 31100475

[140] Park MS Choi S Lee YR Joo HK Kang G Kim CS. Secreted APE1/Ref-1 Inhibits TNF-α-Stimulated Endothelial Inflammation *via* Thiol-Disulfide Exchange in TNF Receptor. Sci Rep (2016) 6:1–12. doi: 10.1038/srep23015 26964514 PMC4786854

[141] Choi S Lee YR Park MS Joo HK Cho EJ Kim HS. Histone Deacetylases Inhibitor Trichostatin A Modulates the Extracellular Release of APE1/Ref-1. Biochem Biophys Res Commun (2013) 435(3):403–7. doi: 10.1016/j.bbrc.2013.04.101 23665318

[142] Lee YR Kim KM Jeon BH Choi S. Extracellularly Secreted APE1/Ref-1 Triggers Apoptosis in Triple-Negative Breast Cancer Cells *via* RAGE Binding, Which is Mediated Through Acetylation. Oncotarget (2015) 6(27):23383–98. doi: 10.18632/oncotarget.4345 PMC4695125 26125438

[143] Lee YR Joo HK Lee EO Cho HS Choi S Kim CS. ATP Binding Cassette Transporter A1 is Involved in Extracellular Secretion of Acetylated APE1/Ref-1. Int J Mol Sci (2019) 20:3178. doi: 10.3390/IJMS20133178 PMC6651529 31261750

[144] Sengupta S Mantha AK Song H Roychoudhury S Nath S Ray S. Elevated Level of Acetylation of APE1 in Tumor Cells Modulates DNA Damage Repair. Oncotarget (2016) 7:75197–209. doi: 10.18632/oncotarget.12113 PMC5342734 27655688

[145] Wilson DM Kim D Berquist BR Sigurdson AJ. Variation in Base Excision Repair Capacity. Mutat Res - Fundam Mol Mech Mutagen (2011) 711:100–12. doi: 10.1016/j.mrfmmm.2010.12.004 PMC3101302 21167187

[146] Hadi MZ Coleman MA Fidelis K Mohrenweiser HW Wilson DM. Functional Characterization of Ape1 Variants Identified in the Human Population. Nucleic Acids Res (2000) 28:3871. doi: 10.1093/NAR/28.20.3871 11024165 PMC110798

[147] Hung RJ Hall J Brennan P Boffetta P. Genetic Polymorphisms in the Base Excision Repair Pathway and Cancer Risk: A Huge Review. Am J Epidemiol (2005) 162:925–42. doi: 10.1093/aje/kwi318 16221808

[148] Negrini S Gorgoulis VG Halazonetis TD. Genomic Instability–an Evolving Hallmark of Cancer. Nat Rev Mol Cell Biol (2010) 11:220–9. doi: 10.1038/NRM2858 20177397

[149] Lirussi L Antoniali G D’Ambrosio C Scaloni A Nilsen H Tell G. APE1 Polymorphic Variants Cause Persistent Genomic Stress and Affect Cancer Cell Proliferation. Oncotarget (2016) 7:26293–306. doi: 10.18632/oncotarget.8477 PMC5041981 27050370

[150] Pieretti M Khattar NH Smith SA. Common Polymorphisms and Somatic Mutations in Human Base Excision Repair Genes in Ovarian and Endometrial Cancers. Mutat Res - Mutat Res Genomics (2001) 432:53–9. doi: 10.1016/S1383-5726(00)00002-9 11465542

[151] Illuzzi JL Harris NA Manvilla BA Kim D Li M Drohat AC. Functional Assessment of Population and Tumor-Associated APE1 Protein Variants. PloS One (2013) 8:1–12. doi: 10.1371/journal.pone.0065922 PMC3679070 23776569

[152] Liu J Zheng B Li Y Yuan Y Xing C. Genetic Polymorphisms of DNA Repair Pathways in Sporadic Colorectal Carcinogenesis. J Cancer (2019) 10:1417–33. doi: 10.7150/jca.28406 PMC6485219 31031852

[153] Tian J Liu G Zuo C Liu C He W Chen H. Genetic Polymorphisms and Gastric Cancer Risk: A Comprehensive Review Synopsis From Meta-Analysis and Genome-Wide Association Studies. Cancer Biol Med (2019) 16:361–76. doi: 10.20892/j.issn.2095-3941.2018.0290 PMC6713634 31516756

[154] Ji YN Zhan P Wang J Qiu LX Yu LK. APE1 Asp148Glu Gene Polymorphism and Lung Cancer Risk: A Meta-Analysis. Mol Biol Rep (2011) 38:4537–43. doi: 10.1007/s11033-010-0584-2 21132382

[155] da Silva TA Fontes FL Coutinho LG Soares de Souza FR Araujo de Melo JT de Souto JT. SNPs in DNA Repair Genes Associated to Meningitis and Host Immune Response. Mutat Res Mol Mech Mutagen (2011) 713(1–2):39–47. doi: 10.1016/j.mrfmmm.2011.05.012 21651918

[156] Whitaker AM Stark WJ Flynn TS Freudenthal BD. Molecular and Structural Characterization of Disease-Associated APE1 Polymorphisms. DNA Repair (Amst) (2020) 91–92:102867. doi: 10.1016/j.dnarep.2020.102867 PMC7295089 32454397

[157] Kay J Thadhani E Samson L Engelward B. Inflammation-Induced DNA Damage, Mutations and Cancer. DNA Repair (Amst) (2019) 83:1–21. doi: 10.1016/j.dnarep.2019.102673 PMC6801086 31387777

[158] Hanahan D Weinberg RA. Hallmarks of Cancer: The Next Generation. Cell (2011) 144:646–74. doi: 10.1016/J.CELL.2011.02.013 21376230

[159] Meira LB Bugni JM Green SL Lee CW Pang B Borenshtein D. DNA Damage Induced by Chronic Inflammation Contributes to Colon Carcinogenesis in Mice. J Clin Invest (2008) 118:2516–25. doi: 10.1172/JC135073 PMC2423313 18521188

[160] Bardia A Tiwari SK Gunisetty S Anjum F Nallari P Habeeb MA. Functional Polymorphisms in XRCC-1 and APE-1 Contribute to Increased Apoptosis and Risk of Ulcerative Colitis. Inflammation Res (2012) 61:359–65. doi: 10.1007/s00011-011-0418-2 22193858

[161] Lin C Jin Y Cheng S Wang W. Association Between APE1 ASP148GLU and Colorectal Cancer Risk: A Meta-Analysis. Clin Invest Med (2020) 43:E24–34. doi: 10.25011/cim.v43i4.34987 33370522

[162] Hinz JM Mao P McNeill DR Wilson DM. Reduced Nuclease Activity of Apurinic/Apyrimidinic Endonuclease (APE1) Variants on Nucleosomes: Identification of Access Residues. J Biol Chem (2015) 290:21067–75. doi: 10.1074/jbc.M115.665547 PMC4543664 26134573

[163] Offer SM Pan-Hammarström Q Hammarström L Harris RS. Unique DNA Repair Gene Variations and Potential Associations With the Primary Antibody Deficiency Syndromes igAD and CVID. PloS One (2010) 5:1–10. doi: 10.1371/journal.pone.0012260 PMC2923613 20805886

[164] Doss CGP NagaSundaram N. Investigating the Structural Impacts of I64T and P311S Mutations in APE1-DNA Complex: A Molecular Dynamics Approach. PloS One (2012) 7:1–11. doi: 10.1371/journal.pone.0031677 PMC3288039 22384055

[165] Olkowski ZL. Mutant AP Endonuclease in Patients With Amyotrophic Lateral Sclerosis. Neuroreport (1998) 9:239–42. doi: 10.1097/00001756-199801260-00012 9507962

[166] McCauley ME Baloh RH. Inflammation in ALS/FTD Pathogenesis. Acta Neuropathol (2019) 137:715–30. doi: 10.1007/s00401-018-1933-9 PMC6482122 30465257

[167] Farg MA Konopka A Soo KY Ito D Atkin JD. The DNA Damage Response (DDR) is Induced by the C9orf72 Repeat Expansion in Amyotrophic Lateral Sclerosis. Hum Mol Genet (2017) 26:2882–96. doi: 10.1093/HMG/DDX170 28481984

[168] Montaldi AP Godoy PRDV Sakamoto-hojo ET. Mutagenesis APE1/REF-1 Down-Regulation Enhances the Cytotoxic Effects of Temozolomide in a Resistant Glioblastoma Cell Line. Mutat Res - Genet Toxicol Environ Mutagen (2015) 793:19–29. doi: 10.1016/j.mrgentox.2015.06.001 26520369

[169] Liuzzi M Talpaert-Borle M. A New Approach to the Study of the Base-Excision Repair Pathway Using Methoxyamine. J Biol Chem (1985) 260:5252–8. doi: 10.1016/s0021-9258(18)89014-7 2580833

[170] Liuzzi M Weinfeld M Paterson MC. Selective Inhibition by Methoxyamine of the Apurinic/Apyrimidinic Endonuclease Activity Associated With Pyrimidine Dimer-DNA Glycosylases From Micrococcus Luteus and Bacteriophage T4. Biochemistry (1987) 26(12):3315–21. doi: 10.1021/bi00386a011 2443160

[171] Chu L Anderson AKL Landers MA Wang Y Kelley MR Messmann RA. CTC Enumeration and Characterization as a Pharmacodynamic Marker in the Phase I Clinical Study of APX3330, an APE1/Ref-1 Inhibitor, in Patients With Advanced Solid Tumors. J Clin Oncol (2019) 37(15_suppl):e14531. doi: 10.1200/jco.2019.37.15_suppl.e14531

[172] Shahda S Lakhani NJ O’Neil B Rasco DW Wan J Mosley AL. A Phase I Study of the APE1 Protein Inhibitor APX3330 in Patients With Advanced Solid Tumors. J Clin Oncol (2019) 37(15_suppl):3097. doi: 10.1200/jco.2019.37.15_suppl.3097

[173] Nanji AA Tahan SR. Association Between Endothelial Cell Proliferation and Pathologic Changes in Experimental Alcoholic Liver Disease. Toxicol Appl Pharmacol (1996) 140:101–7. doi: 10.1006/taap.1996.0202 8806875

[174] Nagakawa J-I Hishinuma I Miyamoto K Hirota K Abe S Yamanaka T. ProtectiveEffectsof(2E)-3-[5-(2,3-Dimethoxy-6-Methyl-1,4- Benzoquinoyl)]-2-Nonyl-2-PropenoicAcidonEndotoxin-Mediated HepatitisinMice. J Pharmacol Exp Ther (1992) 262:145–50. 1625194

[175] Malaguarnera L. Influence of Resveratrol on the Immune Response. Nutrients (2019) 11:1–24. doi: 10.3390/nu11050946 PMC6566902 31035454

[176] Yang S Irani K Heffron SE Jurnak F Meyskens FL. Alterations in the Expression of the Apurinic/Apyrimidinic Endonuclease-1/Redox Factor-1 (APE/Ref-1) in Human Melanoma and Identification of the Therapeutic Potential of Resveratrol as an APE/Ref-1 Inhibitor. Mol Cancer Ther (2005) 4:1923–35. doi: 10.1158/1535-7163.MCT-05-0229 16373707

[177] Pinheiro DML de Oliveira AHS Coutinho LG Fontes FL de Medeiros Oliveira RK Oliveira TT. Resveratrol Decreases the Expression of Genes Involved in Inflammation Through Transcriptional Regulation. Free Radic Biol Med (2019) 130:8–22. doi: 10.1016/j.freeradbiomed.2018.10.432 30366059

[178] Li H Zhong C Wang Q Chen W Yuan Y. Curcumin is an APE1 Redox Inhibitor and Exhibits an Antiviral Activity Against KSHV Replication and Pathogenesis. Antiviral Res (2019) 167:98–103. doi: 10.1016/j.antiviral.2019.04.011 31034848 PMC7388702

[179] Sarkar B Dhiman M Mittal S Mantha AK. Curcumin Revitalizes Amyloid Beta (25–35)-Induced and Organophosphate Pesticides Pestered Neurotoxicity in SH-SY5Y and IMR-32 Cells *via* Activation of APE1 and Nrf2. Metab Brain Dis (2017) 32:2045–61. doi: 10.1007/s11011-017-0093-2 28861684

[180] Zaky A Bassiouny A Farghaly M El-Sabaa BM. A Combination of Resveratrol and Curcumin is Effective Against Aluminum Chloride-Induced Neuroinflammation in Rats. J Alzheimer’s Dis (2017) 60:S221–2. doi: 10.3233/JAD-161115 28222524

[181] Singh-Gupta V Joiner MC Runyan L Yunker CK Sarkar FH Miller S. Soy Isoflavones Augment Radiation Effect by Inhibiting APE1/ref-1 DNA Repair Activity in non-Small Cell Lung Cancer. J Thorac Oncol (2011) 6:688–98. doi: 10.1097/JTO.0b013e31821034ae 21325978

[182] Singh-Gupta V Zhang H Banerjee S Kong D Raffoul JJ Sarkar FH. Radiation-Induced HIF-1α Cell Survival Pathway Is Inhibited by Soy Isoflavones in Prostate Cancer Cells. Int J Cancer (2009) 124:1675–84. doi: 10.1002/ijc.24015 PMC2670478 19101986

[183] Singh-Gupta V Zhang H Yunker CK Ahmad Z Zwier D Sarkar FH. Daidzein Effect on Hormone Refractory Prostate Cancer *In Vitro* and *In Vivo* Compared to Genistein and Soy Extract: Potentiation of Radiotherapy. Pharm Res (2010) 27:1115–27. doi: 10.1007/s11095-010-0107-9 20309614

[184] Liu GD Xia L Zhu JW Ou S Li MX He Y. Genistein Alleviates Radiation-Induced Pneumonitis by Depressing Ape1/Ref-1 Expression to Down-Regulate Inflammatory Cytokines. Cell Biochem Biophys (2014) 69:725–33. doi: 10.1007/s12013-014-9859-x 24659138

